# A Pair of 2D Quantum Liquids: Investigating the Phase Behavior of Indirect Excitons

**DOI:** 10.1021/acsnano.2c06947

**Published:** 2022-09-07

**Authors:** Paul R. Wrona, Eran Rabani, Phillip L. Geissler

**Affiliations:** † Department of Chemistry, University of California, Berkeley, California 94720, United States; ‡ Materials Sciences Division, Lawrence Berkeley National Laboratory, Berkeley, California 94720, United States; ¶ The Raymond and Beverly Sackler Center of Computational Molecular and Materials Science, Tel Aviv University, Tel Aviv 69978, Israel

**Keywords:** electron−hole liquid, indirect exciton, coupled quantum wells, phase transition, Mott transition

## Abstract

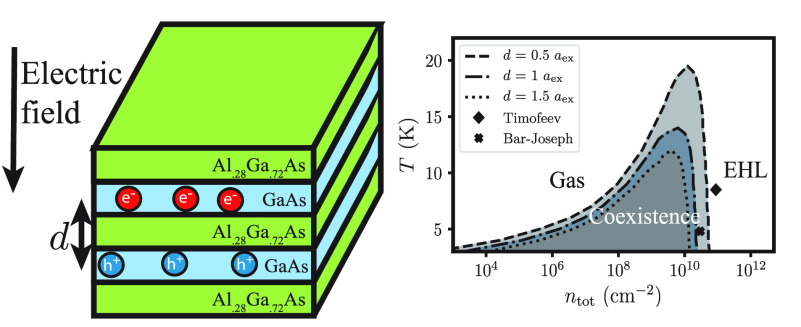

Long-lived indirect excitons (IXs) exhibit a rich phase diagram, including a Bose–Einstein condensate (BEC), a Wigner crystal, and other exotic phases. Recent experiments have hinted at a “classical” liquid of IXs above the BEC transition. To uncover the nature of this phase, we use a broad range of theoretical tools and find no evidence of a driving force toward classical condensation. Instead, we attribute the condensed phase to a quantum electron–hole liquid (EHL), first proposed by Keldysh for direct excitons. Taking into account the association of free carriers into bound excitons, we study the phase equilibrium between a gas of excitons, a gas of free carriers, and an EHL for a wide range of electron–hole separations, temperatures, densities, and mass ratios. Our results agree reasonably well with recent measurements of GaAs/AlGaAs coupled quantum wells.

## Introduction

Excitons are bound states of an electron and a hole attracted to each other by the screened electrostatic Coulomb force, resulting in neutral quasiparticles that can exist in a variety of semiconducting and insulating materials. Their lifetime is determined by the rate of decay to the ground state, either radiatively by emitting photons or nonradiatively by coupling to lattice phonons or other carriers via Auger recombination leading to exciton–exciton annihilation. Understanding these relaxation pathways has been key in the development of light-harvesting devices under low and high photon fluences.

**Figure 1:**
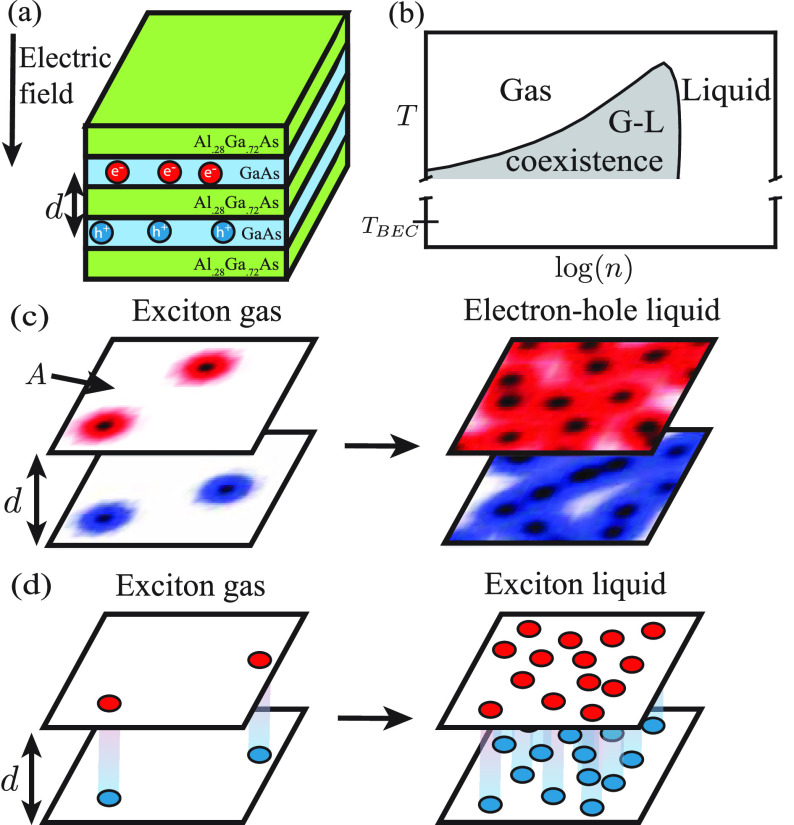
(a) Schematic of coupled quantum wells with the center-to-center distance *d* shown. (b) Sketch of a phase diagram showing the ordering of *T*_BEC_ and *T*_C_, the liquid–gas critical temperature for the transition observed by Bar-Joseph and co-workers. (c) Sketch of a phase transition from a gas of excitons to a degenerate electron–hole liquid. The area of each two-dimensional (2D) plane is *A*. (d) Sketch of a phase transition from a gas of bound indirect excitons to a classical liquid.

The interactions among excitons can also result in a wide variety of thermodynamic phases. At high densities, excitons undergo a Mott transition to an electron–hole plasma stabilized by strong screening effects.^1^ At lower temperatures where quantum statistics dominate, the Mott transition is further facilitated by the favorable exchange interaction between like particles. Additionally, excitons may be regarded as weakly interacting neutral bosons^2^ and can thus form Bose–Einstein condensates^3−5^ (BECs) and superfluid phases.^6^ However, the transience of excitons often complicates experimental realization of such quantum phases. After reaching thermal equilibrium, excitons eventually recombine (radiatively or nonradiatively), preventing further study of their phase behavior. To prevent fast recombination of excitons, recent work has focused on indirect excitons (IXs), whose constituent carriers are confined to two parallel wells that are extended in two directions, due to either an electric field^1^ or type-II band alignment^8^ (see Figure 1a). By restricting the carriers to different regions, IX recombination lifetimes are extended by orders of magnitude, providing a platform to better understand the phase behavior of excitons.

The interactions and collective behavior of indirect excitons differ significantly from their direct counterparts due to the permanent dipole moments they acquire through spatial separation of electrons and holes.^9^ Theoretical studies of such dipolar fluids have revealed quantum and classical phases governed by intriguing correlation regimes.^10−14^ Indeed, several experimental studies have provided evidence for the formation of BECs of IXs at very low temperatures, typically below 1 K.^7,15^ More recently, Bar-Joseph and his co-workers studied the collective behavior of IXs in GaAs/AlGaAs coupled quantum wells (CQWs) over a wider range of temperatures. Above the BEC temperature *T*_BEC_ ≈ 1.1 K but below a critical temperature *T*_C_ = 4.8 K, the excitons separated into two phases (see Figure 1b) distinguished by a several-fold difference in exciton density, i.e., a gas and a liquid, and characterized by a low-energy feature in the photoluminescence spectrum (the Z-line).^16^ Based on previous theoretical work,^10,11^ they argued that the liquid phase results from the repulsive interaction between the dipolar excitons, which generate short-range correlations typical of a “classical” liquid. In a subsequent study,^17^ they concluded that the classical liquid is dark and the appearance of the Z-line in the photoluminescence spectrum is not due to recombination of excitons in the liquid phase, but rather, recombination of excitons in the gas phase near the interface with the liquid.^17^ A dark exciton liquid was also observed by Rapaport and co-workers,^18^ consisting of electrons and holes in parallel spin configurations that cannot couple to light. Despite significant progress in our understanding of the phase behavior of IXs, the origin of the stability of the higher temperature classical liquid still remains unclear.

In this work, we revisit the putative classical liquid phase of excitons. We seek to understand the nature of the liquid phase and the leading correlations that stabilize it at temperatures above *T*_BEC_ and below *T*_C_. In particular, we thoroughly assess whether a dense fluid of IXs, characterized by short-range correlations due to interexciton interactions, can coexist with a much more dilute gas of IXs at equilibrium. The abrupt condensation implied by such a coexistence scenario is unlikely for spatially direct excitons, whose strong tendency to pair up generates at appreciable density a population of weakly interacting biexcitons, akin to a collection of diatomic hydrogen molecules that condense only at very low temperature. The transversely aligned dipoles of IXs, however, inhibit the formation of “excitonic molecules” for large electron–hole separations where the exciton–exciton potential is purely repulsive. However, at moderate separations, this potential is attractive, raising the possibility that IXs condense through van der Waals-like interactions while remaining distinct—neither paired as biexcitons nor strongly influenced by quantum degeneracy. The first part of our work examines this possibility by computing effective interaction potentials for pairs and triads of IXs, with approaches adapted from standard methods of quantum chemistry. Based on these calculations, we conclude that, for experimentally relevant values of the electron–hole separation, excitons are repulsive species that lack an adhesive force that could drive classical condensation.

The second part of our work explores an alternative interpretation of the liquid phase of IXs observed in experiments. It is well known that a gas of spatially direct excitons can condense to form a degenerate electron–hole liquid (EHL), a plasma stabilized by spatial correlations in charge density, as first proposed by Keldysh.^19^ Experiments in the following decades revealed interesting properties of this state, such as high mobility and simple mechanical control through applied stress.^20−23^ This condensed state exists at temperatures low enough to achieve degeneracy, but not low enough to exhibit coherent phenomena or form a BEC. Whether this EHL can account for the liquid phase of IXs is our second main focus. For many different semiconductors, the critical temperature of Keldysh’s EHL can be approximated by *T*_C_ ≈ 0.1*E*_ex_/*k*_B_, where *E*_ex_ is the binding energy of the exciton and *k*_B_ is Boltzmann’s constant. To estimate *E*_ex_, one can model IXs with a bilayer geometry shown in Figure 1c: electrons and holes are placed on two infinitely thin parallel planes separated by *d*. We expect that the neglected out-of-plane fluctuations of the carriers are weak due to spatial confinement. For the experimental setup of Bar-Joseph,^16,17^ this model suggests a critical temperature of *T*_C_ = 3.5 K in comparison to the experimental value of *T*_C_ = 4.8 K. This rough agreement suggests that this phase could be Keldysh’s EHL realized in a bilayer geometry, as sketched in Figure 1c. To study the Keldysh EHL phase and its dependence on the separation between electrons and holes, we adopt a Green’s function approach and approximate the in-plane charge density fluctuations using the random phase approximation (RPA). We find that the Keldysh EHL is stable across a surprisingly wide range of planar separations, supporting the existence of a liquid of dissociated IXs that features strong screening and exchange interactions, rather than a classical liquid stabilized by cohesive forces between charge-neutral excitons.

## Results and Discussion

### Classical Liquid

Condensation of a classical fluid is typically driven by attractive interactions among its constituent particles. Purely repulsive interactions generate very high pressure at high particle densities; matching this pressure in a coexisting phase, as required for thermodynamic equilibrium, is difficult to achieve in a much more dilute state. A fluid of repulsive particles can of course undergo structural phase transitions, as famously exemplified by the crystallization of hard spheres. But coexisting phases of repulsive isometric particles are typically very similar in density, differing more prominently in symmetry or composition in the case of mixtures. Our scrutiny of the classical condensation hypothesis for IXs is thus principally a search for attractive interactions that could plausibly stabilize a dense phase of otherwise repulsive dipolar particles at moderate pressure.

We define an effective two-body interaction potential *V*_ex–ex_(*R*_ex–ex_) as the energy of two interacting excitons separated by a distance *R*_ex–ex_ minus the energy of two noninteracting excitons (−2*E*_ex_). This requires a Born–Oppenheimer-like approximation, in essence taking *R*_ex–ex_ to be fixed while averaging over quantum fluctuations in the excitons’ internal structure. Justifying this simplification requires that the hole is much more (or much less) massive than its partner electron. Our calculation of excitonic interaction potentials will therefore assume infinitely massive holes. In materials of interest, the electron–hole mass ratio σ = *m*_e_/*m*_h_ is not nearly so extreme. The heavy-hole limit (σ = 0) we consider nonetheless provides a useful assessment, as it represents the most favorable scenario for attraction among excitons.

**Figure 2:**
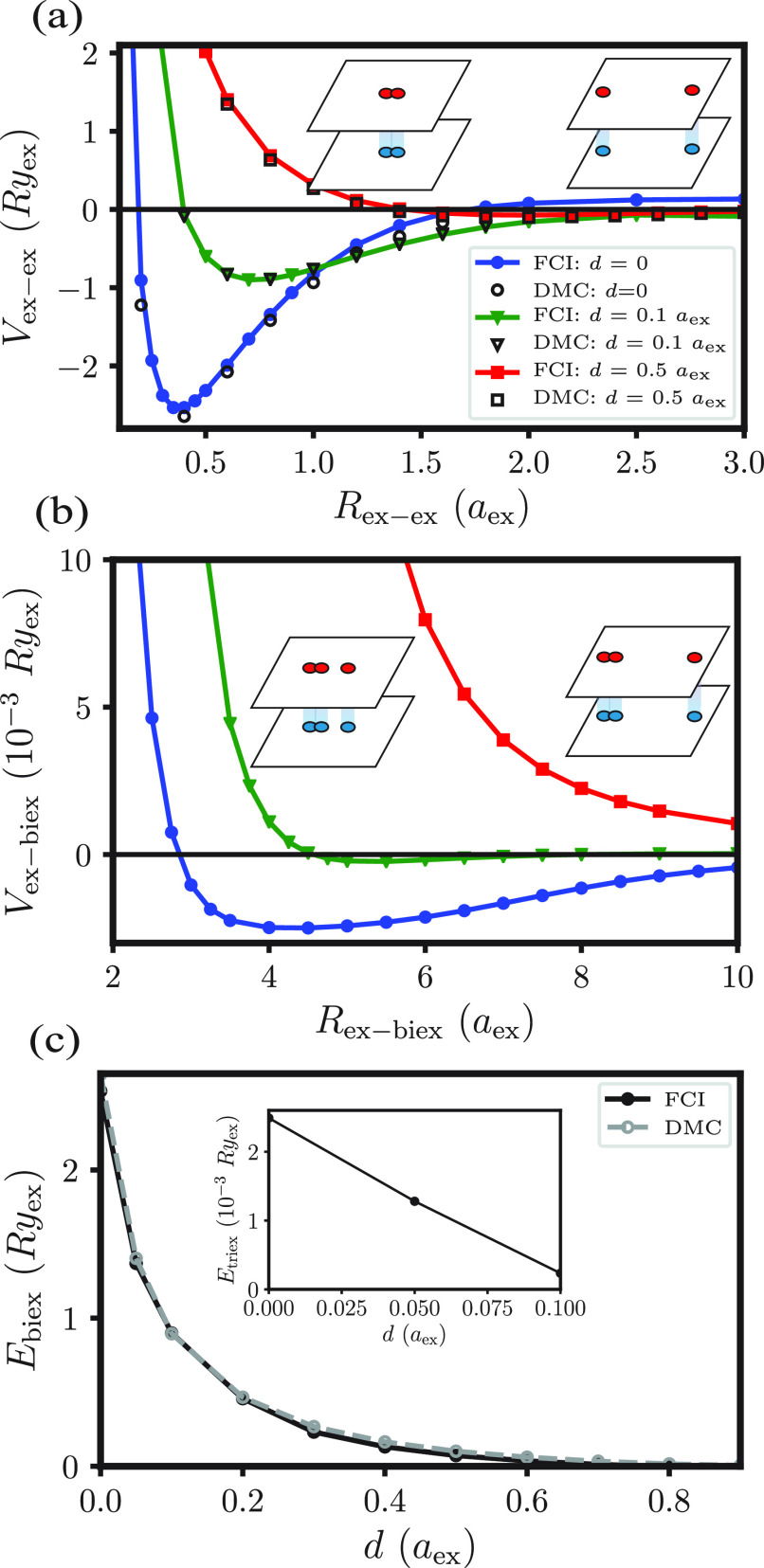
(a) Interaction potentials between two excitons with infinitely heavy holes for various bilayer separations, *d*. We fix the orbitals’ sizes as we pull apart the excitons, so we do not correctly describe dissociation. (See SI for further details.) “FCI” data came from our full CI method, and “DMC” data were computed using the CASINO program.^25^ (b) Interaction potentials between an exciton and a biexciton in a collinear geometry with infinitely heavy holes for various *d*, computed using our FCI method. *R*_ex–biex_ is the distance between the biexciton’s center of mass and the third exciton. (c) Comparison of diffusion Monte Carlo results for the binding energy *E*_biex_ of a biexciton against this work’s FCI method. The inset shows *E*_triex_, the binding energy of a triexciton.

Interactions between a pair of IXs have been computed by Needs and co-workers^24^ using diffusion Monte Carlo (DMC) methods for the same bilayer Hamiltonian we consider. Their results reveal a two-body attraction that weakens rapidly with increasing separation *d*. To serve as a basis for classical condensation, this attraction would need to be additive; that is, a similarly favorable energy would need to be realized as a third exciton is added, then a fourth, and so on. DMC is not well suited for evaluating this additivity, since the electron/hole wave function acquires nodal surfaces when *N* > 2. We instead adopt a configuration interaction (CI) approach, improving systematically on a Hartree–Fock-like mean field approximation, just as in highly accurate quantum chemistry calculations. The SI describes our full CI method in detail, which assumes infinite hole mass and employs a suitable localized basis set. To demonstrate its accuracy, we show in Figure 2a computed pair potentials *V*_ex–ex_ for several bilayer separations, together with DMC results computed using the CASINO program.^25^ While attraction between IXs remains evident at *d* = 0.5*a*_ex_, the biexciton binding energy is a small fraction of *Ry*_ex_ at this separation. Near *d* = 0.8*a*_ex_ the minimum of the interaction potential becomes too shallow to resolve, and for significantly larger *d* the pair potential is purely repulsive.

For *d* = 0, a hydrogenic analogy suggests that the exciton pair attraction represents a kind of covalent bond, with substantial sharing of electron density. As with diatomic hydrogen, we then expect that interactions between this biexciton and additional excitons are noncovalent in character and thus considerably weaker. The exciton–biexciton potential *V*_ex–biex_, plotted in Figure 2b, verifies this expectation. A van der Waals-like attraction favors distances much larger than the “covalent bond” length, and the scale of attractive energy is smaller by 3 orders of magnitude. The same is true for *d* = 0.1*a*_ex_, despite dipolar repulsion that might be imagined to inhibit exciton pairing. For *d* ≥ 0.2*a*_ex_ the effective potential *V*_ex–biex_ exhibits no minimum at all. The results for the biexction and triexciton binding energies as a function of the interlayer separation are summarized in Figure 2c.

The attraction previously demonstrated between exciton pairs is thus not at all additive. Once paired, excitons experience at most extremely weak forces of cohesion. Experimentally relevant bilayer separations *d* > 0.5*a*_ex_ entirely negate attractions involving biexcitons, casting doubt on the classical condensation picture. There remains the possibility that the repulsion among excitons’/biexcitons’ dipoles generates correlations that stabilize liquid–gas phase coexistence.^10,26^ We tested this notion by performing classical Monte Carlo simulations of particles in two dimensions that repel at long-range with energy ∼*R*^–3^ and additionally exclude volume at close range. (See SI for details.) Computed isotherms manifest freezing transitions at high density and pressure but otherwise show no sign of thermodynamic bistability that could be associated with fluid condensation.

### Quantum Liquid

Turning to Keldysh’s degenerate electron–hole liquid, we begin by considering the zero-temperature limit and focus on describing the relative stability of the EHL compared to the exciton gas. Finite temperature effects, including dissociation of bound excitons into an electron–hole gas, will be described below. The total energy per electron for *N* electrons and *N* holes is given by the sum of kinetic, exchange, capacitor, and correlation terms: *E*_tot_ = *E*_kin_ + *E*_exch_ + *E*_cap_ + *E*_corr_, where
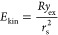
1, and the dimensionless interparticle spacing, *r*_s_, is determined by the relation^27^
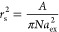
2where *A* is the surface area depicted in Figure 1. (See SI for all details.) In all of the calculations reported below, we take the thermodynamic limit, where *N* → ∞ and *A* → ∞, such that the number density, *n*_tot_ = *N*/*A*, remains a constant. The exchange energy is given exactly by^28^
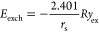
3The capacitor contribution (i.e., the classical electrostatic cost of separating uniformly charged plates by a perpendicular distance *d*) can be written as
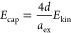
4Finally, the correlation energy in atomic units (ℏ = 1) is estimated within the random phase approximation:
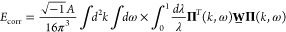
5where λ is the coupling constant, **Π**^*T*^(*k*, ω) = [Π_e_(*k*, ω), Π_h_, (ω)], and Π_*i*_(*k*, ω) is the 2D Lindhard polarizability for particle *i* = e,*h* evaluated at wavevector *k* and frequency ω. Within the RPA, the screened Coulomb matrix is given by
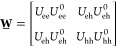
6where *U*_*ij*_(λ, *k*, ω) is the effective interaction between particles *i* and *j* and *U*_*ij*_^0^(λ, *k*) is the corresponding bare Coulomb interaction in *k*-space. These quantities and the full details of RPA calculations are further described in the SI.

**Figure 3:**
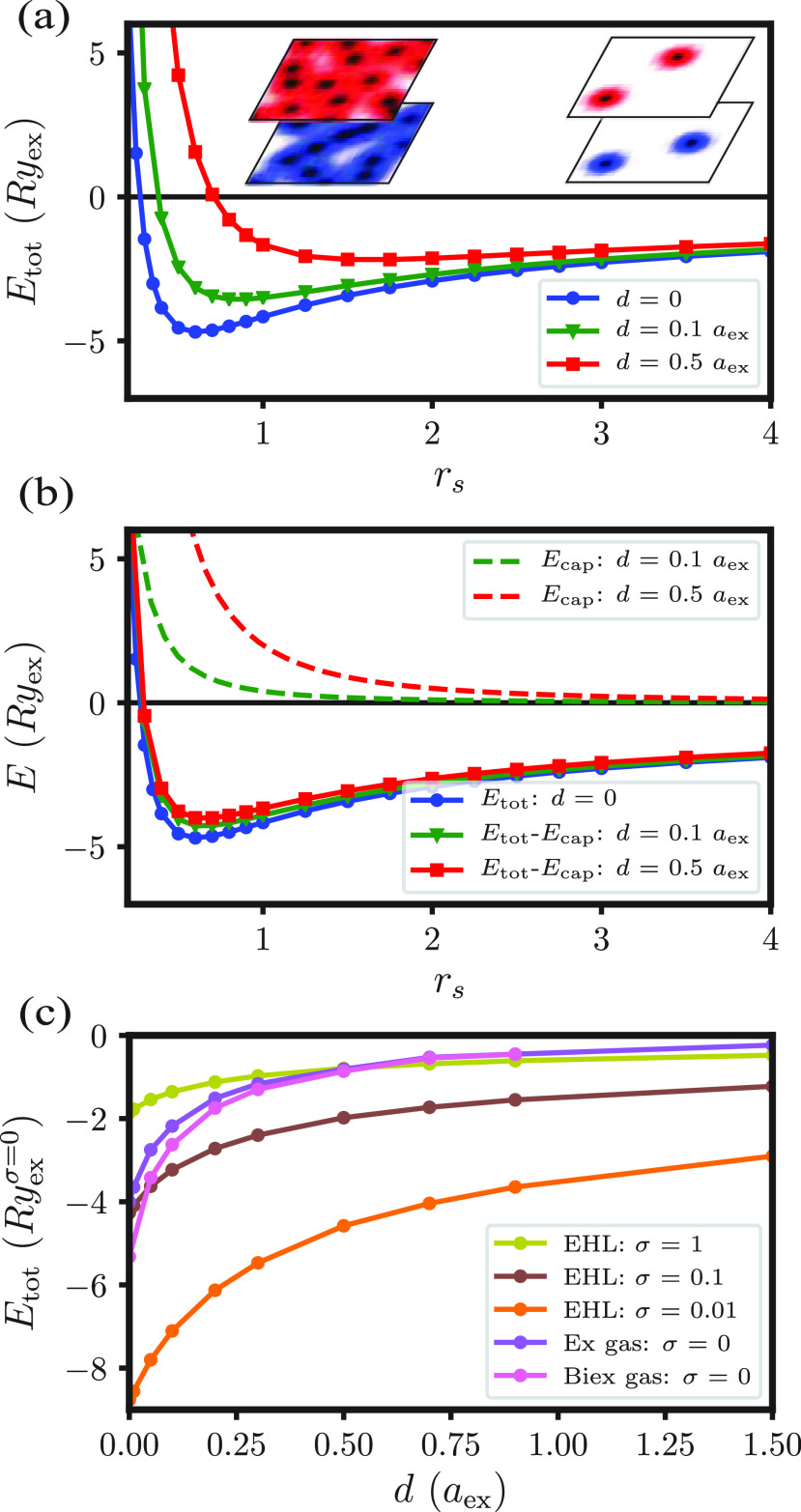
(a) Total energy per electron of an EHL with σ = 0.1 as a function of the average interparticle spacing, *r*_s_, evaluated for various *d*. (b) The total energies shown in (a) minus the capacitor term (shown in dashed lines) as a function of the average interparticle spacing. (c) Minimum energy of an EHL with σ = 1, 0.1, and 0.01. For the case σ = 0, we also plot the energy of an exciton (“Ex”) gas at the same charge density as the EHL, and similarly for a biexciton (“Biex”) gas.

In Figure 3a, we plot the resulting total energy per electron *E*_tot_ as a function of *r*_s_ (eq 2) for three different bilayer separations and for a mass ratio σ = *m*_e_/*m*_h_ = 0.1. We find that the total energy shows a pronounced minimum, *r*_s,eq_, near *a*_ex_ for small bilayer separations, signifying the existence of a stable degenerate electron–hole liquid. The major contribution to the change in the total energy as the bilayer separation increases is the capacitor term; without this term, results for different *d* are nearly identical, as shown in Figure 3b. We note that the total energy of spatially separated electrons and holes has been calculated previously in the superfluid^29^ and superconducting^30^ regimes using a Green’s function and variational approach, respectively. In both cases, the *d*-dependence on the energy agrees with our results.

The minimum energies of an EHL with σ = 1, 0.1, and 0.01 are shown in Figure 3c. We also present the energies of a gas of excitons and gas of biexcitons. To compare these states on equal footing, we add to their energies the capacitor term evaluated at *r*_s,eq_. Ignoring the possibility of a BEC phase, we find that for most values of *d*, the EHL is the stable phase at zero temperature and high densities. Specifically, for σ = 0.1 and *d* = 1.5*a*_ex_, we find that the total energy for carriers in the EHL is larger (i.e., more negative) than the energy of the IX gas by approximately 1 *Ry*_ex_. This value agrees with the observations of Bar-Joseph and co-workers,^16^ who measured a ∼1 *Ry*_ex_ energy shift in their photoluminescence spectra between the IX gas and the condensed phase.

Next we turn to the effect of thermal fluctuations on the relative stability of IX gas and EHL phases. In doing so, it is important to acknowledge that the gas phase is not devoid of free charge carriers, nor is the liquid devoid of bound excitons. Instead, their proportions in each phase are determined by a chemical equilibrium e^–^ + h^+^ ⇌ X that requires the chemical potential μ_X_ of an exciton to equal that of an unbound electron–hole pair, μ_eh_. We treat interactions involving excitons as purely electrostatic and mean-field, giving μ_X_ = *k*_B_*T* ln(1 – exp[−*n*_X_λ_X_^2^/ξ_X_]) – *E*_ex_ + μ_cap_, where the first term is an ideal contribution for bosons in two dimensions, *n*_X_ is the excitons’ density, λ_X_ is their thermal de Broglie wavelength, ξ_X_ = 4 is their spin degeneracy, and *E*_X_ is their binding energy. The capacitor potential, μ_cap_ = 4π*de*^2^*n*_tot_, depends only on *d* and the total density *n*_tot_ = *n*_X_ + *n*_eh_ of excitations. The free carrier chemical potential,
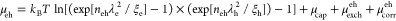
7includes an ideal contribution for the Fermionic species (ξ_e_ = ξ_h_ = 2), the capacitor potential, exchange effects from both electrons and holes, and a correlation term obtained from a generalization of RPA to finite temperature^31^ (see SI for details). We thus obtain a law of mass action for the fraction α = *n*_eh_/*n*_tot_ of carriers that are not bound as excitons (eq 8)):
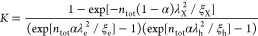
8where
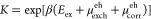
9For very low density (*n*_tot_ ≪ λ_e_^–2^), effects of quantum statistics become unimportant, and eq 8 reduces to the Saha ionization equation, a classical law of mass action. At densities typical of the degenerate EHL, quantum statistical effects are essential.

**Figure 4:**
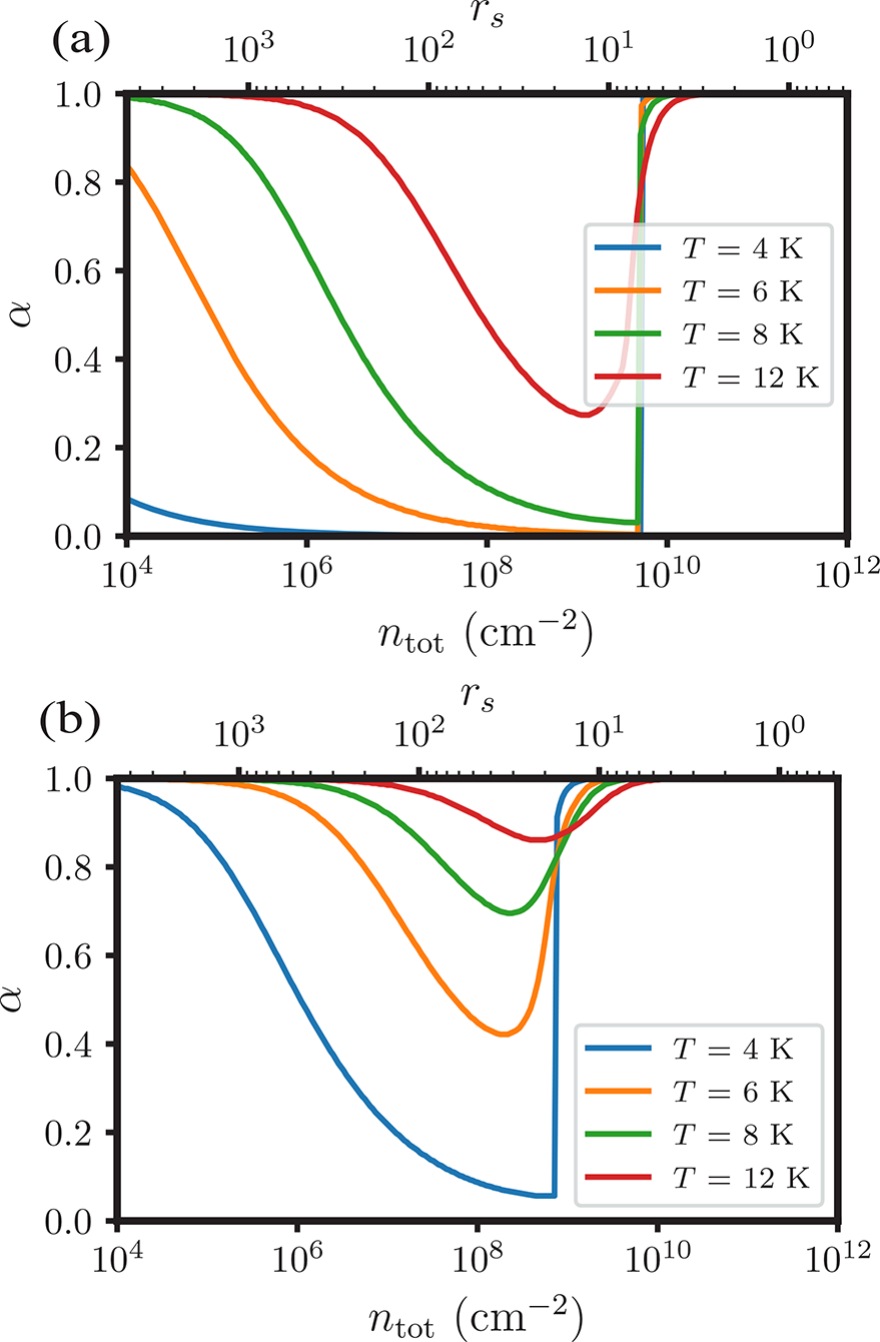
Fraction of free carriers, α, as a function of the total density *n*_tot_ (in units of excitations per cm^–2^) for an electron–hole system with σ = 0.1 at various temperatures. Results for *d* = 0.5*a*_ex_ are shown in (a) and for *d* = 1.5*a*_ex_ in (b).

For a spatially uniform density *n*_tot_ of excitations, solving eq 8 gives the fraction α of free carriers at thermal equilibrium. In Figure 4 we plot α as a function of *n*_tot_ for (a) *d* = 0.5*a*_ex_ and (b) *d* = 1.5*a*_ex_ for several temperatures, using parameters appropriate for GaAs. In the very dilute gas, α ≈ 1 – (constant)*n*e^–β*E*_ex_^ ≈ 1, since exciton dissociation is strongly favored by the entropy of mixing. With increasing density, α decreases steadily due to the favorable energy of exciton binding, until exchange and correlation become dominant at high density. *K* rapidly approaches zero as a result, yielding a very small population of neutral excitons. Under some conditions the increase in free carrier fraction at high density occurs discontinuously, a result of eq 8 acquiring multiple roots. (We select the root that minimizes the total free energy, as detailed in the SI.) This abrupt change in conductivity at density *n*_Mott_(*d*, *T*) signals a first-order exciton Mott transition. It can occur only below a critical temperature *T*_Mott_(*d*), as evident in Figure 4(b) for *d* = 1.5*a*_ex_ where *T*_Mott_ ≈ 4 K. As the exciton binding energy *E*_ex_ and EHL correlation energy *E*_corr_ decline in magnitude with increasing bilayer separation, *T*_Mott_ also decreases.

**Figure 5:**
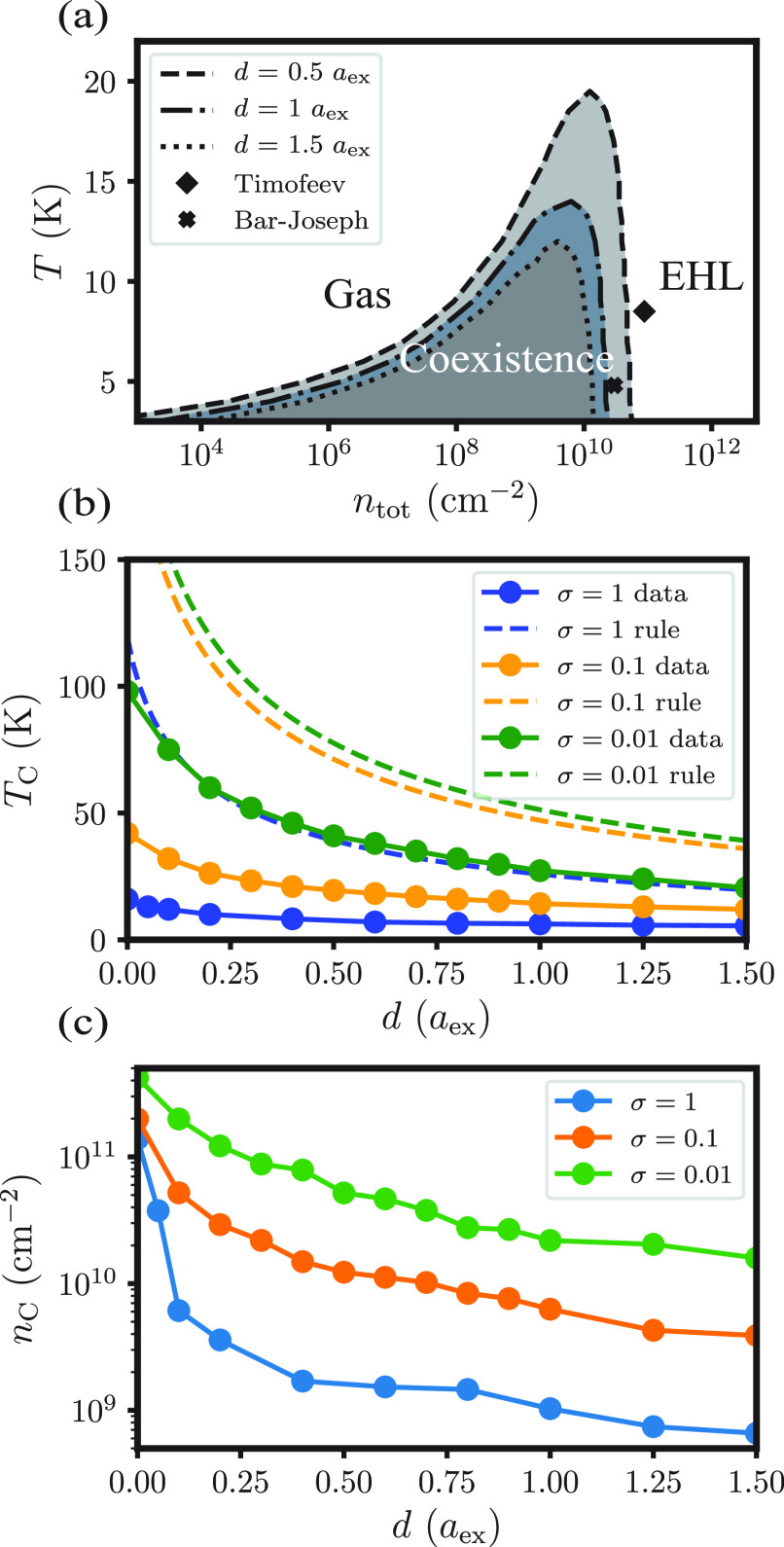
(a) Phase diagrams in the density–temperature plane for EHL condensation with σ = 0.1 at various bilayer separations *d*. Experimentally estimated critical temperatures are shown for *d* = 1*a*_ex_ (“Timofeev” data^32^) and for *d* = 1.5*a*_ex_ (“Bar-Joseph” data^1,17^). The total density *n*_tot_ has units of excitations per cm^–2^. (b) Computed critical temperature as a function of bilayer separation *d* for various mass ratios σ. Also shown is the empirical rule *T*_C_ ≈ 0.1*E*_ex_/*k*_B_. (c) Computed critical (total) density as a function of bilayer separation *d* for various mass ratios σ.

The assumption of spatial uniformity, however, may break down before the Mott transition is encountered, and we find that this is in fact the case. The function μ_eh_(*n*_tot_) we obtain by combining eq 7 with the law of mass action develops an instability at low temperature. Specifically, (∂μ_eh_/∂*n*_tot_)_*T*_ < 0 over a range of intermediate densities, violating thermodynamic stability criteria and implying a phase-separated equilibrium state. We determine this state of coexistence—typically featuring a low-density gas enriched in IXs and a liquid of predominantly free carriers—from μ_eh_(*n*_tot_) using a standard Maxwell equal-area construction (see SI for details). Figure 5a shows the resulting gas–EHL phase diagrams for three bilayer separations, each of which exhibits a first-order condensation transition below a critical temperature *T*_C_(*d*) . In each case, *T*_C_ exceeds *T*_Mott_, and the coexisting densities straddle *n*_Mott_(*d*, *T*) . States with uniform density *n*_Mott_(*d*, *T*) are therefore unstable with respect to phase separation, and the first-order Mott transition described above is superseded by condensation.

With increasing bilayer separation, the critical excitation density *n*_C_ and temperature *T*_C_ both systematically decrease, as shown in Figure 5b and c. The empirical formula *T*_C_ ≈ 0.1*E*_ex_/*k*_B_ anticipates this lowering of *T*_C_ due to the weakening exciton binding energy. We find that the phase diagrams for *d* > 0 can be well approximated simply by adding the *d*-dependent capacitor term μ_cap_ to the chemical potentials calculated for *d* = 0 in addition to using the correct value of *E*_ex_. Separating electrons and holes into distinct quantum wells thus appears to influence EHL condensation predominantly through a classical electrostatic bias, disfavoring dense-excitation states due to the necessity of separating substantial charge.

Figure 5a also includes experimental data for GaAs, for which σ = 0.1 and ϵ = 12.9. Beyond predicting the general decrease in *T*_C_ and *n*_C_ with increasing *d*, our estimates of the critical temperature are within a factor of 2–3 of the experimental data. While our predictions of *n*_C_ are off by an order of magnitude, we note that experimental measurements of the critical density often rely on mean-field or steady-state approximations and depend on many different parameters, such as the gate voltage. A material with different dielectric properties would set a different energy scale for electron–hole binding and screening, and the temperatures in Figure 5 would be scaled accordingly. A change in the mass ratio σ has more subtle effects, but basic trends in *n*_C_ and *T*_C_ can be anticipated with the same reasoning used to explain EHL stability at zero temperature. Because a very massive hole serves to localize electrons, we expect stabilization of the dense liquid phase with decreasing σ < 1 at fixed reduced mass (and similarly for increasing σ > 1). Correspondingly, *n*_C_ and *T*_C_ should both increase as σ deviates from unity, as we observe and show in Figure 5b and c.

## Conclusions

In summary, our approximate treatment of a simplified model for interacting electrons and holes in coupled quantum wells yields a low-temperature phase diagram that agrees reasonably well with experimental results. Given the assumptions we have made (a single band, effective masses, and a structureless isotropic background) and the experimental challenges of measuring a precise critical density and temperature, we consider the level of agreement to be a strong suggestion that the liquid phase observed in the laboratory has the same basic character as that in our model. Even for *d* > 1*a*_ex_, the model’s condensed phase is unambiguously a variant of Keldysh’s electron–hole liquid: a degenerate plasma of strongly screened charge carriers and very few bound excitons. By contrast, our calculation of effective interaction potentials among bound electron–hole pairs strongly discourages the notion of a classical liquid comprised of intact excitons as the equilibrium state at the temperatures and densities of interest. Cohesive forces that stabilize biexcitons weaken considerably as the bilayers separate, but they nonetheless dwarf any attraction to a third exciton. At *d* = 1 *a*_ex_ the interactions we compute are purely repulsive and cannot support phase coexistence between a sparse gas and dense liquid of excitons. The strong evidence for a stable Keldysh liquid of IXs and the predictions made for how the critical behavior changes with *d* await experimental validation.

## Methods

We analyze an idealized Hamiltonian *Ĥ* for a collection of *N* electron–hole pairs in coupled quantum wells based on a single-band effective mass approximation. The total Hamiltonian in atomic units (ℏ = *m*_0_ = *e* = 4πϵ_0_ = 1, where ℏ is the reduced Planck constant, *m*_0_ is the electron’s rest mass, *e* is the elementary charge, and ϵ_0_ is the vacuum permittivity), reads
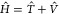
10where the kinetic energy is given by
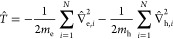
11and *m*_e_ (*m*_h_) is the electron (hole) effective mass. Interactions among charge carriers are described by a screened Coulomb potential:
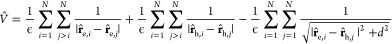
12where ϵ is the static dielectric constant of the material. Excitonic units are used throughout this paper, with energies expressed relative to the exciton Rydberg *Ry*_ex_ = *m*_red_*e*^4^/(2(4*πϵ*_0_ϵ)^2^ℏ^2^) and lengths relative to the exciton Bohr radius *a*_ex_ = 4πϵ_0_ϵℏ^2^/(*m*_red_*e*^2^), where *m*_red_^–1^ = *m*_e_^–1^ + *m*_h_^–1^ is the electron–hole reduced mass.

## Supporting Information Available

Derivation of single-particle basis sets; details of the FCI calculations; pressure–density isotherms for classical dipolar particles in two dimensions; derivation of contributions to the zero-temperature ground-state energy and to the Helmholtz free energy; fitting procedure for exchange–correlation free energies; procedure for solving the law of mass action; procedure for Maxwell equal-area constructions (PDF)

## Notes

This work was previously submitted to a preprint server.^33^
