# Supplementary material for: A Pair of 2D Quantum Liquids: Investigating the Phase Behavior of Indirect Excitons
Source: ACS Nano. 2022 Sep 7;16(9):15339–46. doi: 10.1021/acsnano.2c06947 (PMC9527805; doi:10.1021/acsnano.2c06947)
Supplement: Supplementary file 1 — nn2c06947_si_001.pdf [file nn2c06947_si_001.pdf]

Supporting Information: A Pair of 2D Quantum Liquids: Investigating the Phase Behavior of Indirect Excitons

Paul R. Wrona,*,† Eran Rabani,*,†,‡,¶ and Phillip L. Geissler*,†,‡

†*Department of Chemistry, University of California, Berkeley, California 94720, USA*

‡*Materials Sciences Division, Lawrence Berkeley National Laboratory, Berkeley, California 94720, USA*

¶*The Raymond and Beverly Sackler Center of Computational Molecular and Materials Science, Tel Aviv University, Tel Aviv 69978, Israel*

E-mail: pwrona2@berkeley.edu; eran.rabani@berkeley.edu; geissler@berkeley.edu

Classical liquid

Single particle basis set

Our single particle basis was the set of solutions to the time-independent Schrödinger equation for an exciton in a bilayer geometry (shown in Fig. 1c in the main text). Within the single-band isotropic effective mass approximation and in relative coordinates,

$$\Big(-\frac{1}{2m_{\text{red}}}\nabla^2 - \frac{1}{\epsilon\sqrt{r^2+d^2}}\Big)\phi(r,\theta) = E\phi(r,\theta). \tag{S1}$$

The electron-hole reduced mass is $m_{\text{red}}^{-1} = m_{\text{e}}^{-1} + m_{\text{h}}^{-1}$, ϵ is the static dielectric constant, and d is the bilayer separation. After separating variables, $\phi_{n,m}(r,\theta) = R_{n,m}(r)\Theta_m(\theta)$, the

angular component is

$$\Theta_m(\theta) = \frac{1}{\sqrt{2\pi}} \exp[im\theta] \tag{S2}$$

where m is the angular quantum number and the radial component is the solution to

$$-\frac{1}{2m_{\text{red}} r} \frac{d}{dr}\big(r \frac{dR_{n,m}}{dr}\big) + \big(\frac{m^2}{2m_{\text{red}} r^2} - \frac{1}{\epsilon\sqrt{r^2 + d^2}}\big) R_{n,m}(r) = E_{n,m} R_{n,m}(r) \tag{S3}$$

where n is the principal quantum number. We solved the radial equation on a real-space grid using the Arnoldi method to obtain the first several radial wavefunctions $R_{n,m}(r)$. We approximated these functions by Gaussians or products of Gaussians and polynomials of x and y as is typical for Gaussian basis sets.[S1] For $m \neq 0$, these functions are not peaked at the origin so we first remove a factor of r^m:

$$f(r) = r^{-m} R(r). \tag{S4}$$

The resulting functions f were then approximated by a linear combination of Gaussians by choosing coefficients c_i and variances σ_i^2 to minimize

$$\int_0^\infty r\big[f(r) - \sum_{i=1}^{N_g} c_i g(r, \sigma_i^2)\big]^2 dr \tag{S5}$$

using the BFGS algorithm.[S2] We found that five Gaussians ($N_g = 5$) yields an error less than 1% for all states up to and including $n = 4$.

Full configuration interaction calculations for biexcitons

Defining an exciton-exciton interaction potential required us to invoke a Born-Oppenheimer-like approximation as discussed in the main text. Thus, we assumed the holes were infinitely massive point particles on one plane and sought the electronic states located on the other plane. We used the full configuration interaction (FCI) method and solved the generalized

eigenvalue problem

$$HC = SC\varepsilon. \tag{S6}$$

H is the Hamiltonian matrix corresponding to the biexciton Hamiltonian (Eqs. (1)-(3) in the main text with $N = 2$), C is the matrix of coefficients, S is the overlap matrix, and ε is the vector of biexciton energies E_{XX}.

Our FCI biexciton wavefunction was the product of an asymmetric singlet spin function and the following symmetric spatial component:

$$\Psi_{\text{biex}}(\mathbf{r}_1, \mathbf{r}_2) = \sum_{i,j=1}^{N_{\text{orbitals}}} c_{ij}\big(\phi_i(\mathbf{r}_1)\phi_j(\mathbf{r}_2) + \phi_j(\mathbf{r}_1)\phi_i(\mathbf{r}_2)\big). \tag{S7}$$

$\mathbf{r}_1$ is the position of electron 1, ϕ_i is an orbital characterized by the quantum numbers n and m and centered directly above one of the holes, *et cetera.* The exciton-exciton interaction potential was defined as

$$V_{\text{ex}-\text{ex}}(R_{\text{ex}-\text{ex}}) = E_{\text{XX}}(R_{\text{ex}-\text{ex}}) - 2E_{\text{ex}} \tag{S8}$$

where $R_{\text{ex}-\text{ex}}$ is the separation between the two holes and E_{ex} is the energy of an indirect exciton within our basis. When we used "scaled" orbitals (discussed below), the exciton energy was evaluated using these scaled orbitals.

Fig. S1 shows exciton-exciton interaction potentials for $d = 0$ using various single particle orbitals. As we increased our basis size N_{orbitals}, both E_{ex} and E_{XX} decreased. However, the latter energy almost always decreased more quickly, so $V_{\text{ex}-\text{ex}}$ decreased as our basis set improved in general. Our basis set is complete in theory so we would recover the exact (*i.e.*, DMC) result if we use an infinite number of orbitals. However, as seen in Fig. S1 the convergence rate is rather slow. Inspired by Dunning's correlation-consistent basis sets,[S3] we optimized the size of the exciton orbitals in order to minimize E_{XX} more quickly. Specifically, we divided σ_i, the standard deviations of the Gaussians used to construct these orbitals, by

"scaling factors" in order to minimize E_{XX} at the equilibrium exciton-exciton separation R_{eq} and subsequently fixed these scaled orbitals for all exciton-exciton separations. Because we did not allow the orbitals to relax as the excitons were separated from one another, we did not describe dissociation properly. While this introduced an artificial long-range repulsion, the most important feature of these interaction potentials was the inter-exciton attraction. Table S1 contains the scaling factors used to optimize the exciton orbitals in addition to the values of R_{eq} for all bilayer separations under consideration.

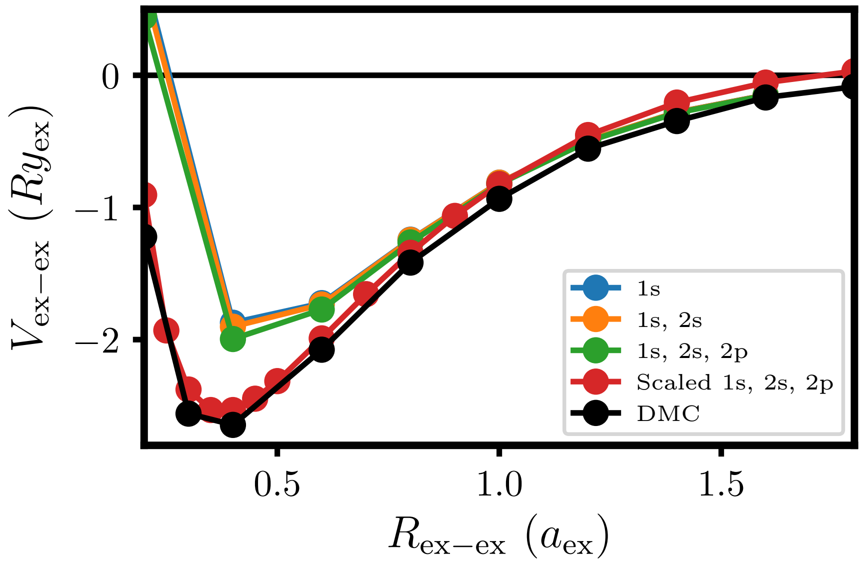

Figure S1: Exciton-exciton interaction potentials for $d = 0$. The legend contains the various single particle states used in the FCI calculations. The potentials converge towards the "exact" result (DMC) as the basis increases and improves in quality.

Full configuration interaction calculations for triexcitons

We computed triexciton energies E_{XXX} in a manner similar to the biexciton case. The ground state spin configuration for three electrons is a doublet. To derive the corresponding spatial

Table S1: Optimal scaling factors for our Gaussian basis sets.

d (a_{ex})	1s factor	$2p_x$ factor	$2p_y$ factor	2s factor	R_{eq} (a_{ex})
0	1.3	0.8	1.0	4.6	0.4
0.05	1.2	0.6	5.0	3.7	0.55
0.1	1.1	3.8	4.1	3.2	0.7
0.2	1.1	3.1	3.4	2.7	1.0
0.3	1.0	2.5	2.8	2.5	1.3
0.4	1.0	2.3	2.5	2.2	1.7
0.5	1.0	2.1	2.3	2.0	2.0
0.6	1.0	2.0	2.2	1.9	2.3
0.7	1.0	1.9	2.1	1.7	2.7
0.8	1.0	1.8	1.9	1.5	3.1
0.9	1.0	1.7	1.8	1.3	3.8

wavefunction, we started from the following Slater determinant:

$$\Psi_{XXX}(\mathbf{r}_1, \mathbf{r}_2, \mathbf{r}_3, \omega_1, \omega_2, \omega_3) = \begin{vmatrix} \phi_a(\mathbf{r}_1)\alpha(\omega_1) & \phi_b(\mathbf{r}_1)\beta(\omega_1) & \phi_c(\mathbf{r}_1)\alpha(\omega_1) \\ \phi_a(\mathbf{r}_2)\alpha(\omega_2) & \phi_b(\mathbf{r}_2)\beta(\omega_2) & \phi_c(\mathbf{r}_2)\alpha(\omega_2) \\ \phi_a(\mathbf{r}_3)\alpha(\omega_3) & \phi_b(\mathbf{r}_3)\beta(\omega_3) & \phi_c(\mathbf{r}_3)\alpha(\omega_3) \end{vmatrix} \tag{S9}$$

where ϕ_a is a single particle spatial orbital, α and β are spin functions, and ω_i is the spin variable for electron i. Without loss of generality, we paired the spins of electrons 1 and 2 into a singlet state and coupled the spin of the third electron which we chose to be up. In the uncoupled basis, we have

$$|\text{doublet}\rangle = \Big(\frac{|\uparrow\downarrow\rangle_{1,2} - |\downarrow\uparrow\rangle_{1,2}}{\sqrt{2}}\Big)\,|\uparrow\rangle_3 = \frac{|\uparrow\downarrow\uparrow\rangle_{1,2,3} - |\downarrow\uparrow\uparrow\rangle_{1,2,3}}{\sqrt{2}} \tag{S10}$$

Since singlet states have zero spin, we know that in the coupled basis,

$$|\text{doublet}\rangle = |\frac{1}{2}; +\frac{1}{2}\rangle \tag{S11}$$

Finally, we projected Eq. (S9) onto Eq. (S10) to obtain

$$
\begin{aligned}
&|\frac{1}{2};+\frac{1}{2}\rangle\left(\frac{\langle\uparrow\downarrow\uparrow|-\langle\downarrow\uparrow\uparrow|}{\sqrt{2}}\right)\begin{vmatrix}\phi_a(\mathbf{r}_1)\,|\uparrow\rangle & \phi_b(\mathbf{r}_1)\,|\downarrow\rangle & \phi_c(\mathbf{r}_1)\,|\uparrow\rangle\\ \phi_a(\mathbf{r}_2)\,|\uparrow\rangle & \phi_b(\mathbf{r}_2)\,|\downarrow\rangle & \phi_c(\mathbf{r}_2)\,|\uparrow\rangle\\ \phi_a(\mathbf{r}_3)\,|\uparrow\rangle & \phi_b(\mathbf{r}_3)\,|\downarrow\rangle & \phi_c(\mathbf{r}_3)\,|\uparrow\rangle\end{vmatrix}\\
&=\Big(\phi_a(\mathbf{r}_1)\phi_b(\mathbf{r}_2)\phi_c(\mathbf{r}_3)-\phi_c(\mathbf{r}_1)\phi_b(\mathbf{r}_2)\phi_a(\mathbf{r}_3)\\
&-\phi_b(\mathbf{r}_1)\phi_c(\mathbf{r}_2)\phi_a(\mathbf{r}_3)+\phi_b(\mathbf{r}_1)\phi_a(\mathbf{r}_2)\phi_c(\mathbf{r}_3)\Big)\frac{1}{\sqrt{2}}\,|\frac{1}{2};+\frac{1}{2}\rangle
\end{aligned}
\tag{S12}
$$

Summing over all possible orbitals, the FCI triexciton wavefunction reads

$$
\begin{aligned}
\Psi_{\text{triex}}(\mathbf{r}_1,\mathbf{r}_2,\mathbf{r}_3)=\sum_{i,j}^{N_{\text{orbitals}}}\sum_{k\neq i}^{N_{\text{orbitals}}}&\Big(\phi_i(\mathbf{r}_1)\phi_j(\mathbf{r}_2)\phi_k(\mathbf{r}_3)+\phi_j(\mathbf{r}_1)\phi_i(\mathbf{r}_2)\phi_k(\mathbf{r}_3)\\
&-\phi_k(\mathbf{r}_1)\phi_j(\mathbf{r}_2)\phi_i(\mathbf{r}_3)-\phi_j(\mathbf{r}_1)\phi_k(\mathbf{r}_2)\phi_i(\mathbf{r}_3)\Big)
\end{aligned}
\tag{S13}
$$

After computing triexciton energies, the exciton-biexciton interaction potential was computed according to

$$
V_{\text{ex}-\text{biex}}(R_{\text{ex}-\text{biex}};R_{\text{ex}-\text{ex}})=E_{\text{XXX}}(R_{\text{ex}-\text{biex}})-E_{\text{ex}}-E_{\text{XX}}(R_{\text{ex}-\text{ex}})
\tag{S14}
$$

$R_{\text{ex}-\text{ex}}$ is the separation between the two closest excitons which we found to be almost exactly equal to R_{eq} in the energy-minimizing geometry. $R_{\text{ex}-\text{biex}}$ is the separation between the biexciton's center-of-mass and the furthest exciton.

Thermodynamics of dipolar particles in two dimensions

Using computer simulations, we explored the possibility that quasiparticle correlations due to long-range dipolar repulsion can drive condensation of a classical liquid in 2D. Specifically, we sampled particle configurations r^N from a Boltzmann distribution $P(r^N)\propto e^{-\beta U(r^N)}$ with

$U = (1/2)\sum_i \sum_{j\neq i} u(r_{ij})$. The pair interaction potential

$$u(r) = 4\epsilon_{\mathrm{LJ}}\left[\left(\frac{\sigma}{r}\right)^{12} - \left(\frac{\sigma}{r}\right)^{6}\right] + \frac{A}{r^3},$$

where A is a positive constant, adds dipolar repulsion to the standard Lennard-Jones potential. Taking ϵ_{LJ} and σ as units of energy and length, respectively, the dimensionless potential becomes

$$u(r) = 4\left(r^{-12} - r^{-6}\right) + \frac{A^*}{r^3},$$

where $A^* = A/(\epsilon_{\mathrm{LJ}}\sigma^3)$ sets the relative strength of dipolar repulsion. For $A^* \gtrsim 2.2$ the classical potential $u(r)$ is repulsive at all r. For $A^* \lesssim 2.2$, $u(r)$ has a local minimum near contact, and for $A^* \lesssim 1.6$ this contact minimum becomes globally stable. The range $1.5 < A^* < 2.5$ thus spans the range of potentials we have computed using FCI methods.

To establish an appropriate range of temperature for this model system, we estimate the energy of dipolar repulsion near contact for a coupled quantum well. In a bilayer geometry, a pair of excitons (each with the electron perfectly aligned over the hole) separated by lateral distance r has Coulomb interaction energy

$$u_{\mathrm{Coulomb}}(r) = 2\mathrm{Ry}_{\mathrm{ex}}\left[2\frac{a_{\mathrm{ex}}}{r} - 2\frac{a_{\mathrm{ex}}}{\sqrt{r^2+d^2}}\right]$$

which gives a dipolar repulsion to lowest order in d/r:

$$u_{\mathrm{dipole}}(r) = 2\mathrm{Ry}_{\mathrm{ex}}\frac{(d/a_{\mathrm{ex}})^2}{(r/a_{\mathrm{ex}})^3}$$

Identifying a_{ex} as the rough volume-excluding size of an exciton, and focusing on the case $d = a_{\mathrm{ex}}$ this gives an energy near contact of

$$u_{\mathrm{dipole}}(r_{\mathrm{contact}}) = 2\mathrm{Ry}_{\mathrm{ex}}.$$

Comparing this energy scale to the observed critical temperature in GaAs, $k_{\mathrm{B}}T_{\mathrm{c}} \approx 0.3\mathrm{Ry}_{\mathrm{ex}}$, we have

$$k_{\mathrm{B}}T_{\mathrm{c}} \approx 0.15 u_{\mathrm{dipole}}(r_{\mathrm{contact}})$$

In our simple model, the dipolar energy at contact is simply A/σ^3, giving

$$\frac{k_{\mathrm{B}}T_{\mathrm{c}}}{\epsilon_{\mathrm{LJ}}} \approx 0.15 A^*.$$

For the dipole strengths of interest ($1.5 < A^* < 2.5$), we would anticipate a dimensionless critical temperature in the range $T_{\mathrm{c}} \approx 0.2$ to 0.4 (if condensation were to occur at all).

Using Metropolis Monte Carlo sampling, we have computed pressure-density isotherms at thermodynamic conditions spanning the ranges described above, for a 2D system of $N = 400$ particles. Specifically, we consider three dipole strengths, $A^* = 1.5, 2$, and 2.5. For each of these strengths we consider three temperatures, $T = 0.1, 0.3$, and 0.5. Isotherms are determined for each combination of A^* and T by calculating the average virial pressure at a series of densities, beginning with a dilute gas at reduced density $\rho = 0.05$. The equilibrated state at this density is then compressed to a slightly higher density $\rho' = \rho + \Delta\rho$, with $\Delta\rho = 0.01$. Following an equilibration period of 1000 Monte Carlo sweeps, we compute a new average pressure over the course of 9000 sweeps. The density is increased again by $\Delta\rho$, and equilibration and averaging are repeated as before. This compression protocol is followed until the density reaches $\rho = 0.85$, a tightly packed and highly organized state with the rough appearance of crystalline order (which cannot strictly persist over large scales in two dimensions). We subsequently reduce the density in steps of $\Delta\rho$, computing a new series of average pressures at the same thermodynamic states as in the compression protocol.

Computed isotherms are plotted in Fig. S2. A condensation transition would manifest in these results in two ways. First, it would be accompanied by strong hysteresis, i.e., significant discrepancies between compression and expansion results over a range of densities within the liquid-gas coexistence region, due to high free energy barriers for condensation

and vaporization. Second, the fully equilibrated macroscopic isotherm would feature constant pressure across the broad range of densities between gas and liquid. For our finite system, such nonanalytic behavior would be rounded but still marked by a nearly constant pressure at coexistence. Neither of these features are evident in our results.

Compression and expansion results are essentially indistinguishable at all but a few densities. These small regions of hysteresis occur at high densities characteristic of a solid phase. Given the subtleties of freezing in two dimensions,[S4] we have not attempted to assign the high-density phase as hexatic or crystalline; for our purposes, it is important only that (a) this state is not fluid, and (b) the fluid it coexists with is similarly dense, i.e., not a gas by any reasonable measure. The slight hysteresis we find at high density is thus clearly associated with freezing rather than condensation.

In summary, the signatures of condensation – hysteresis and near-constant pressure between a low gas-like density and a much higher liquid-like density – are entirely absent in our simulation results. We note that a tail correction to the pressure,[S5] resulting from the unavoidable truncation of particle interactions, has not been included. This contribution, proportional to the square of density, cannot give rise to hysteresis and would only exacerbate the observed non-constancy of pressure.

Quantum liquid

Ground state energy at zero temperature

Following previous work on the electron-hole liquid,[S6,S7] the ground state energy per electron at zero temperature was given by

$$E_{\mathrm{tot}} = E_{\mathrm{kin}} + E_{\mathrm{exch}} + E_{\mathrm{cap}} + E_{\mathrm{corr}}. \tag{S15}$$

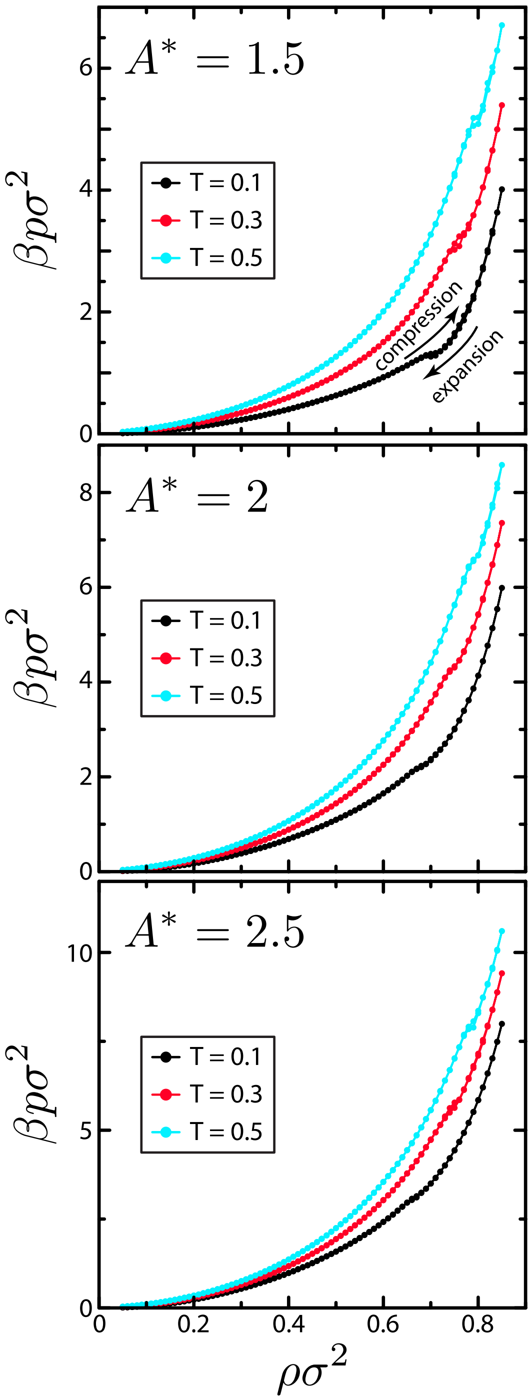

Figure S2: Pressure-density isotherms for a system of dipolar particles in two dimensions. For each dipole strength A^*, simulation results are shown for three different temperatures. Each curve includes data for both compression and expansion protocols. See text for additional numerical details.

Kinetic energy

The total kinetic energy of all electrons and holes was expressed as the sum of the non-interacting energies over all occupied states:

$$\mathcal{E}_{\text{kin}} = \sum_{i=e,h} 2 \sum_{k<k_{\text{F}_i}} \frac{\hbar^2 k^2}{2m_i} \tag{S16}$$

where k_{F_i} is the Fermi wavevector for particle i. In the thermodynamic limit, the sum can be replaced by an integral,[S8] so

$$\mathcal{E}_{\text{kin}} = \sum_{i=\text{e,h}} 2 \frac{\hbar^2}{2m_i} \frac{A}{(2\pi)^2} \int_0^{k_{\text{F}_i}} 2\pi k(k^2) dk \tag{S17}$$

$$= \sum_{i=\text{e,h}} 2 \frac{\hbar^2}{2m_i} \frac{A}{2\pi} \frac{k_{\text{F}_i}^4}{4} \tag{S18}$$

After writing the Fermi wavevectors in terms of the density,

$$\mathcal{E}_{\text{kin}} = \sum_{i=\text{e,h}} 2 \frac{\hbar^2 k_{\text{F}_i}^2}{2m_i} \frac{N}{4} \tag{S19}$$

$$\frac{\mathcal{E}_{\text{kin}}}{N} = E_{\text{kin}} = \hbar^2 \Big(\frac{k_{\text{F}_e}^2}{2m_e} + \frac{k_{\text{F}_\text{h}}^2}{2m_\text{h}} \Big) \tag{S20}$$

Using

$$k_{\text{F}_i} a_{\text{ex}} = \frac{\sqrt{2}}{r_s} \tag{S21}$$

we have

$$E_{\text{kin}} = \frac{Ry_{\text{ex}}}{r_s^2} \tag{S22}$$

Exchange energy

The exchange energy was expressed as

$$\mathcal{E}_{\text{exch}} = -\sum_{i=\text{e,h}} \frac{2}{2A} \sum_{\mathbf{k},\mathbf{p}} U^0(\mathbf{k}-\mathbf{p})\Theta(k-k_{\text{F}_i})\Theta(p-k_{\text{F}_i}) \tag{S23}$$

where the Fourier transform of the statically-screened two-dimensional Coulomb interaction is

$$U^0(k) = \frac{2\pi e^2}{4\pi\epsilon_0\epsilon k}. \tag{S24}$$

After replacing the double sum by a double integral, the expression was evaluated exactly:

$$\frac{\mathcal{E}_{\text{exch}}}{N} = -\sum_{i=e,h} \frac{8\sqrt{2}}{3\pi r_s} Ry_{\text{ex}} \tag{S25}$$

$$E_{\text{exch}} = -\frac{2.401}{r_s} Ry_{\text{ex}} \tag{S26}$$

Capacitor energy

The capacitor energy is the $q = 0$ term of the Fourier transform of the potential energy given in Eq. 3 in the main text. Each individual term in Eq. 3 diverges in the thermodynamic limit yet their sum is finite. We therefore introduced exponential convergence factors to regularize the terms:

$$V = \frac{1}{2}\sum_{i\neq j}^{N} \frac{\exp[-\mu|\mathbf{r}_{e,i}-\mathbf{r}_{e,j}|]}{\epsilon|\mathbf{r}_{e,i}-\mathbf{r}_{e,j}|} + \frac{1}{2}\sum_{i\neq j}^{N} \frac{\exp[-\mu|\mathbf{r}_{h,i}-\mathbf{r}_{h,j}|]}{\epsilon|\mathbf{r}_{h,i}-\mathbf{r}_{h,j}|} - \sum_{i,j}^{N} \frac{\exp[-\mu|\mathbf{r}_{e,i}-\mathbf{r}_{h,j}|]}{\epsilon\sqrt{|\mathbf{r}_{e,i}-\mathbf{r}_{h,j}|^2+d^2}}. \tag{S27}$$

To write this in second quantization, we first evaluated

$$\begin{aligned} \langle \mathbf{k}_1\xi_1\mathbf{k}_2\xi_2|\, V\, |\mathbf{k}_3\xi_3\mathbf{k}_4\xi_4\rangle = \frac{1}{\epsilon A^2}\delta_{\xi_1,\xi_3}\delta_{\xi_2,\xi_4} \int d\mathbf{r}_1 \int d\mathbf{r}_2 \exp[-i(\mathbf{k}_1\cdot\mathbf{r}_1+\mathbf{k}_2\cdot\mathbf{r}_2)] \\ \times \exp[i(\mathbf{k}_3\cdot\mathbf{r}_1+k_4\cdot\mathbf{r}_2)]\Big\{\frac{\exp[-\mu|\mathbf{r}_1-\mathbf{r}_2|]}{|\mathbf{r}_1-\mathbf{r}_2|} - \frac{\exp[-\mu|\mathbf{r}_1-\mathbf{r}_2|]}{\sqrt{|\mathbf{r}_1-\mathbf{r}_2|^2+d^2}}\Big\} \end{aligned} \tag{S28}$$

where ξ_i is the spin variable of particle i. Let $\mathbf{r} = \mathbf{r}_2$ and $\mathbf{y} = \mathbf{r}_1 - \mathbf{r}_2$, so

$$\begin{aligned}\langle \mathbf{k}_1\xi_1\mathbf{k}_2\xi_2| V |\mathbf{k}_3\xi_3\mathbf{k}_4\xi_4\rangle = \frac{1}{\epsilon A^2}\delta_{\xi_1,\xi_3}\delta_{\xi_2,\xi_4}\int d\mathbf{r}\exp[-i(\mathbf{k}_1+\mathbf{k}_2-\mathbf{k}_3-\mathbf{k}_4)\cdot\mathbf{r}] \\ \times\int d\mathbf{y}\exp[i(\mathbf{k}_3-\mathbf{k}_1)\cdot\mathbf{y}]\Big\{\frac{\exp[-\mu y]}{y}-\frac{\exp[-\mu y]}{\sqrt{y^2+d^2}}\Big\}\end{aligned} \tag{S29}$$

The $\mathbf{r}$-integral gave an area times a Kronecker δ-function which guarantees momentum conservation. Let $\mathbf{q} = \mathbf{k}_1 - \mathbf{k}_3$ be the momentum transferred in the two-particle interaction.

$$\begin{aligned}\langle \mathbf{k}_1\xi_1\mathbf{k}_2\xi_2| V |\mathbf{k}_3\xi_3\mathbf{k}_4\xi_4\rangle = \frac{1}{\epsilon A}\delta_{\xi_1,\xi_3}\delta_{\xi_2,\xi_4}\delta_{\mathbf{k}_1+\mathbf{k}_2,\mathbf{k}_3+\mathbf{k}_4} \\ \times\int_0^\infty dy\int_0^{2\pi} d\theta y\exp[-iqy\cos\theta]\Big\{\frac{\exp[-\mu y]}{y}-\frac{\exp[-\mu y]}{\sqrt{y^2+d^2}}\Big\}\end{aligned} \tag{S30}$$

To obtain the capacitor energy, we first address the $q = 0$ case. After evaluating the θ-integral,

$$\begin{aligned}\langle \mathbf{k}_1\xi_1\mathbf{k}_2\xi_2| V |\mathbf{k}_3\xi_3\mathbf{k}_4\xi_4\rangle = \frac{1}{\epsilon A^2}\delta_{\xi_1,\xi_3}\delta_{\xi_2,\xi_4}\delta_{\mathbf{k}_1+\mathbf{k}_2,\mathbf{k}_3+\mathbf{k}_4} \\ \times\int_0^\infty dy\Big\{\exp[-\mu y]-\frac{y\exp[-\mu y]}{\sqrt{y^2+d^2}}\Big\} \quad \text{for } q=0\end{aligned} \tag{S31}$$

Turning to the y-integral, we first considered

$$\int_0^L dy\frac{y}{\sqrt{y^2+d^2}} \tag{S32}$$

(We will take $L \to \infty$ momentarily.) Letting $u = y^2$,

$$\int_0^L dy\frac{y}{\sqrt{y^2+d^2}} = \frac{1}{2}\int_0^{L^2} du\frac{u}{\sqrt{u+d^2}} \tag{S33}$$

$$= \sqrt{u+d^2}\Big|_{u=0}^{u=L^2} \tag{S34}$$

$$= \sqrt{L^2+d^2} - d \tag{S35}$$

$$= L\Big(1+\frac{1}{2}\big(\frac{d}{L}\big)^2+...\Big) - d \tag{S36}$$

In the thermodynamic limit (*i.e.*, large L/d),

$$\int_0^L dy \frac{y}{\sqrt{y^2+d^2}} = L - d \tag{S37}$$

so

$$\lim_{L\to\infty} \int_0^L dy \Big\{ 1 - \frac{y}{\sqrt{y^2+d^2}} \Big\} = d \tag{S38}$$

We added the convergence factor without effect:

$$\lim_{\mu\to 0} \int_0^\infty dy \Big\{ \exp[-\mu y] - \frac{y \exp[-\mu y]}{\sqrt{y^2+d^2}} \Big\} = d \tag{S39}$$

Therefore,

$$\langle \mathbf{k}_1 \xi_1 \mathbf{k}_2 \xi_2 | \, V \, | \mathbf{k}_3 \xi_3 \mathbf{k}_4 \xi_4 \rangle = \frac{2\pi d}{\epsilon A} \delta_{\xi_1,\xi_3} \delta_{\xi_2,\xi_4} \delta_{\mathbf{k}_1+\mathbf{k}_2,\mathbf{k}_3+\mathbf{k}_4} \tag{S40}$$

For the $q \neq 0$ case, we set $\mu = 0$ in Eq. (S30) and introduce a Bessel function of order zero to get

$$\langle \mathbf{k}_1 \xi_1 \mathbf{k}_2 \xi_2 | \, V \, | \mathbf{k}_3 \xi_3 \mathbf{k}_4 \xi_4 \rangle = \frac{2\pi}{\epsilon A} \delta_{\xi_1,\xi_3} \delta_{\xi_2,\xi_4} \delta_{\mathbf{k}_1+\mathbf{k}_2,\mathbf{k}_3+\mathbf{k}_4} \int_0^\infty dy J_0(qy) \times \Big\{ 1 - \frac{y}{\sqrt{y^2+d^2}} \Big\} \quad \text{for } q \neq 0 \tag{S41}$$

$$= \frac{2\pi}{\epsilon A} \delta_{\xi_1,\xi_3} \delta_{\xi_2,\xi_4} \delta_{\mathbf{k}_1+\mathbf{k}_2,\mathbf{k}_3+\mathbf{k}_4} \Big\{ \frac{1}{q} - \frac{\exp[-qd]}{q} \Big\} \tag{S42}$$

Putting everything together and reintroducing the factors of $e^2/(4\pi\epsilon_0\epsilon)$, our second-quantized Coulomb interaction was

$$V = \frac{e^2\pi}{4\pi\epsilon_0\epsilon A} \sum_{\mathbf{k}\neq 0} \sum_{\mathbf{k},\mathbf{p}} \sum_{\xi_1,\xi_2} \Big\{ \frac{1}{q} a^\dagger_{\mathbf{k}+\mathbf{q},\xi_1} a^\dagger_{\mathbf{p}-\mathbf{q},\xi_2} a_{\mathbf{p},\xi_2} a_{\mathbf{k},\xi_1} + \frac{1}{q} b^\dagger_{\mathbf{k},\xi_1} b^\dagger_{\mathbf{p},\xi_2} b_{\mathbf{p}-\mathbf{q},\xi_2} b_{\mathbf{k}+\mathbf{q},\xi_1} - \frac{2\exp[-qd]}{q} a^\dagger_{\mathbf{k}+\mathbf{q},\xi_1} b^\dagger_{\mathbf{p},\xi_2} b_{\mathbf{p}-\mathbf{q},\xi_2} a_{\mathbf{k},\xi_1} \Big\} + \frac{2\pi e^2 N^2 d}{4\pi\epsilon_0\epsilon A} \tag{S43}$$

where the last term is the capacitor energy, $a^\dagger_{\mathbf{k},\xi_i}$ ($a_{\mathbf{k},\xi_i}$) creates (destroys) an electron with

wavevector $\mathbf{k}$ and spin ξ_i, and $b^{\dagger}_{\mathbf{k},\xi_i}$ ($b_{\mathbf{k},\xi_i}$) creates (destroys) a hole with wavevector $\mathbf{k}$ and spin ξ_i.

Correlation energy

For our general ($d \neq 0$) two-component system, the correlation energy was given by[S9]

$$\mathcal{E}_{\rm corr} = \frac{\hbar i A}{2(2\pi)^3}\int_0^1 \frac{d\lambda}{\lambda}\int d^2k \int d\omega \begin{bmatrix} \Pi_e & \Pi_h \end{bmatrix} \begin{bmatrix} U^{\lambda}_{\rm ee}\lambda U^0_{\rm ee} & U^{\lambda}_{\rm eh}\lambda U^0_{\rm eh} \\ U^{\lambda}_{\rm eh}\lambda U^0_{\rm eh} & U^{\lambda}_{\rm hh}\lambda U^0_{\rm hh} \end{bmatrix} \begin{bmatrix} \Pi_e \\ \Pi_h \end{bmatrix} \tag{S44}$$

where

$$U^0_{ij} = \begin{cases} \frac{2\pi e^2}{k} & i = j \\ -\frac{2\pi e^2}{k}\exp[-kd] & i \neq j \end{cases} \tag{S45}$$

and U^{λ}_{ij} are the dynamically-screened effective interactions.

Π_i is the two-dimensional Lindhard polarizability for particle i.[S7] The real part is

$$\mathrm{Re}\Pi_i(k,\omega) = \frac{m_i}{\hbar^2\pi}\Big[-1 - \frac{\omega - k^2}{2k^2}\sqrt{1-(\frac{2k}{\omega - k^2})^2} + \frac{\omega + k^2}{2k^2}\sqrt{1-(\frac{2k}{\omega + k^2})^2}\Big] \tag{S46}$$

and the imaginary part is

$$\mathrm{Im}\Pi_i(k,\omega) = -\frac{m_i}{\hbar^2\pi k}\Big[\sqrt{1-(\frac{\omega - k^2}{2k})^2} - \sqrt{1-(\frac{\omega + k^2}{2k})^2}\Big] \tag{S47}$$

In both expressions, each square root must be set to zero when it acquires a negative argument.

Within the random-phase approximation,[S8] the effective interactions were given by the following Dyson-like or Ornstein-Zernike-like equations

$$U^{\lambda}_{\rm ee} = \lambda U^0_{\rm ee} + \lambda U^0_{\rm ee}\Pi_{\rm e}U^{\lambda}_{\rm ee} + \lambda U^0_{\rm eh}\Pi_{\rm h}U^{\lambda}_{\rm eh} \tag{S48}$$

$$U^{\lambda}_{\rm hh} = \lambda U^0_{\rm hh} + \lambda U^0_{\rm eh}\Pi_{\rm e}U^{\lambda}_{\rm eh} + \lambda U^0_{\rm hh}\Pi_{\rm h}U^{\lambda}_{\rm hh} \tag{S49}$$

$$U_{\rm eh}^{\lambda} = \lambda U_{\rm eh}^{0} + \lambda U_{\rm ee}^{0}\Pi_{\rm e}U_{\rm eh}^{\lambda} + \lambda U_{\rm eh}^{0}\Pi_{\rm h}U_{\rm hh}^{\lambda} \tag{S50}$$

or in matrix form,

$$\begin{bmatrix} U_{\rm ee}^{\lambda} & U_{\rm eh}^{\lambda} \\ U_{\rm eh}^{\lambda} & U_{\rm hh}^{\lambda} \end{bmatrix} = \lambda \begin{bmatrix} U_{\rm ee}^{0} & U_{\rm eh}^{0} \\ U_{\rm eh}^{0} & U_{\rm hh}^{0} \end{bmatrix} + \lambda \begin{bmatrix} \Pi_{\rm e}U_{\rm ee}^{0} & \Pi_{\rm h}U_{\rm eh}^{0} \\ \Pi_{\rm e}U_{\rm eh}^{0} & \Pi_{\rm h}U_{\rm hh}^{0} \end{bmatrix} \begin{bmatrix} U_{\rm ee}^{\lambda} & U_{\rm eh}^{\lambda} \\ U_{\rm eh}^{\lambda} & U_{\rm hh}^{\lambda} \end{bmatrix}. \tag{S51}$$

The correlation energy was rewritten as

$$\begin{aligned} \mathcal{E}_{\rm corr} =& \frac{\hbar i A}{2(2\pi)^3} \int_0^1 \frac{d\lambda}{\lambda} U_{\rm ee}^{0} \int d^2k \int d\omega \begin{bmatrix} \Pi_{\rm e} & \Pi_{\rm h} \end{bmatrix} \\ & \times \begin{bmatrix} U_{\rm ee} & -U_{\rm eh}\exp[-kd] \\ -U_{\rm eh}\exp[-kd] & U_{\rm hh} \end{bmatrix} \begin{bmatrix} \Pi_{\rm e} \\ \Pi_{\rm h} \end{bmatrix} \end{aligned} \tag{S52}$$

The matrix of effective interactions was written in terms of the bare electron-electron interaction. Substitution of this result led to

$$\begin{aligned} \mathcal{E}_{\rm corr} = \frac{\hbar i A}{2(2\pi)^3} \int d^2k \int d\omega \int_0^1 \frac{d\lambda}{\lambda} \frac{(\lambda U_{\rm ee}^{0})^2}{1 - \lambda\big(\Pi_{\rm e} + \Pi_{\rm h}\big)U_{\rm ee}^{0} + \lambda^2\big(1 - \exp[-4kd]\big)\Pi_{\rm e}\Pi_{\rm h}\big(U_{\rm ee}^{0}\big)^2} \\ \times \begin{bmatrix} \Pi_{\rm e} & \Pi_{\rm h} \end{bmatrix} \begin{bmatrix} 1 + \lambda\big(\exp[-4kd] - 1\big)\Pi_{\rm h}U_{\rm ee}^{0} & \exp[-2kd] \\ \exp[-2kd] & 1 + \lambda\big(\exp[-4kd] - 1\big)\Pi_{\rm e}U_{\rm ee}^{0} \end{bmatrix} \begin{bmatrix} \Pi_{\rm e} \\ \Pi_{\rm h} \end{bmatrix} \end{aligned} \tag{S53}$$

To express this in $Ry_{\rm ex}$, we scaled lengths by $a_{\rm ex}$, wavevectors by $k_{\rm F}$, and frequencies by

$\hbar^2 k_\mathrm{F}^2/(2m_\mathrm{red})$. Thus, the correlation energy per electron was

$$E_\mathrm{corr} = \frac{i}{\pi r_s^2}\int_0^\infty dk \int_0^\infty d\omega \int_0^1 d\lambda \frac{k\lambda\big(U_{ee}^0\big)^2}{1-\lambda\big(\Pi_e+\Pi_h\big)U_{ee}^0+\lambda^2\big(1-\exp[-4\sqrt{2}kd/r_s]\big)\Pi_e\Pi_h\big(U_{ee}^0\big)^2}$$
$$\times\begin{bmatrix}\Pi_e & \Pi_h\end{bmatrix}\begin{bmatrix}1+\lambda\big(\exp[-4\sqrt{2}kd/r_s]-1\big)\Pi_h U_{ee}^0 & \exp[-2\sqrt{2}kd/r_s] \\ \exp[-2\sqrt{2}kd/r_s] & 1+\lambda\big(\exp[-4\sqrt{2}kd/r_s]-1\big)\Pi_e U_{ee}^0\end{bmatrix}$$
$$\times\begin{bmatrix}\Pi_e \\ \Pi_h\end{bmatrix} Ry_\mathrm{ex} \tag{S54}$$

The result of the matrix multiplication is

$$\begin{aligned}E_\mathrm{corr} =& \frac{i}{\pi r_s^2}\int_0^\infty dk \int_0^\infty d\omega \int_0^1 d\lambda k \frac{\lambda}{1-\lambda\big(\Pi_\mathrm{e}+\Pi_\mathrm{h}\big)U_\mathrm{ee}^0+\lambda^2\big(1-\exp[-4\sqrt{2}kd/r_s]\big)\Pi_\mathrm{e}\Pi_\mathrm{h}\big(U_\mathrm{ee}^0\big)^2}\\ &\times\Big(\big(U_\mathrm{ee}^0\big)^2\big(\Pi_\mathrm{e}^2+\Pi_\mathrm{h}^2\big)+2\big(U_\mathrm{ee}^0\big)^2\Pi_\mathrm{e}\Pi_\mathrm{h}\exp[-2\sqrt{2}kd/r_s]\\ &+\lambda\big(\exp[-4\sqrt{2}kd/r_s]-1\big)\big(U_\mathrm{ee}^0\big)^3\big(\Pi_\mathrm{e}+\Pi_\mathrm{h}\big)\Pi_\mathrm{e}\Pi_\mathrm{h}\Big)Ry_\mathrm{ex}\end{aligned} \tag{S55}$$

Chemical potentials

The chemical potentials for excitons and free carriers included ideal and capacitor contributions, both of which were evaluated exactly. However, for electrons and holes, there were additional exchange and correlation contributions that were evaluated numerically. In this case, we first evaluated Helmholtz free energies as a function of density, fit these functions to a simple form, and then computed derivatives to obtain the exchange-correlation chemical potentials.

Ideal chemical potentials

The chemical potential for ideal fermions or bosons in two dimensions can be derived starting from the partition function

$$\Xi = \sum_{n_1,n_2,\ldots,n_k,\ldots} \exp\Big[-\beta\sum_k(\varepsilon_k-\mu)n_k\Big] \tag{S56}$$

where n_k are occupation numbers and $\varepsilon_{i,k}$ are the single particle energies given by

$$\varepsilon_{i,k} = \frac{\hbar^2 k^2}{2m_i}. \tag{S57}$$

For fermions, factorizing the exponential in Eq. (S56) gives

$$\Xi = \prod_k \Big\{ \sum_{n_k=0}^{1} \exp\Big[-\beta(\epsilon_k - \mu) n_k \Big] \Big\} \tag{S58}$$

$$= \prod_k \Big\{ 1 + \exp\Big[-\beta(\epsilon_k - \mu) \Big] \Big\} \tag{S59}$$

The total number of particles was given by

$$\langle N \rangle = \frac{\partial \ln \Xi}{\partial(\beta\mu)} \tag{S60}$$

$$= \sum_k \frac{\exp[\beta(\mu - \varepsilon_k)]}{1 + \exp[\beta(\mu - \varepsilon_k)]} \tag{S61}$$

$$= \sum_k f(k, \beta, \mu) \tag{S62}$$

where f is the Fermi-Dirac distribution. Moving to the thermodynamic limit, we replaced the sum by an integral and wrote

$$\langle N \rangle = \frac{\xi_i A}{(2\pi)^2} \int d^2k \frac{\exp[\beta(\mu - \varepsilon_{i,k})]}{1 + \exp[\beta(\mu - \varepsilon_{i,k})]} \tag{S63}$$

$$= \frac{\xi_i A}{2\pi} \int_0^\infty \frac{\exp[\beta(\mu - \varepsilon_{i,k})]}{1 + \exp[\beta(\mu - \varepsilon_{i,k})]} k dk \tag{S64}$$

where $\xi_i = 2$ is the spin degeneracy for particle i. We evaluated the integral using a u-substitution: $u = \exp[\beta(\mu - \varepsilon_{i,k})]$.

$$\langle N_i \rangle = \frac{\xi_i A}{2\pi} \frac{m_i}{\hbar^2 \beta} \int_0^{\exp[\beta\mu]} \frac{1}{1+y} dy \tag{S65}$$

$$= \frac{\xi_i A}{\lambda_i^2} \ln\big(1 + \exp[\beta\mu]\big) \tag{S66}$$

where we have the thermal de Broglie wavelength

$$\lambda_i^2 = \frac{2\pi\hbar^2\beta}{m_i} \tag{S67}$$

After some algebra, we obtained the chemical potential for non-interacting fermions in two dimensions:

$$\beta\mu_0^i = \ln\big(\exp[n\lambda_i^2/(\xi_i)] - 1\big). \tag{S68}$$

For bosons, the grand partition function was written as

$$\Xi = \prod_k \Big(1 - \exp[\beta(\mu - \varepsilon_k)]\Big)^{-1} \tag{S69}$$

Following the same steps as above, we get

$$\langle N_i \rangle = \xi_i \sum_k \frac{\exp[\beta(\mu - \varepsilon_{i,k})]}{1 - \exp[\beta(\mu - \varepsilon_{i,k})]} \tag{S70}$$

$$= \frac{\xi_i A}{2\pi} \frac{m_i}{\hbar^2\beta} \int_0^{\exp[\beta\mu]} \frac{1}{1-y} dy \tag{S71}$$

$$= -\frac{\xi_i A}{\lambda_i^2} \ln\big(1 - \exp[\beta\mu]\big) \tag{S72}$$

Hence, we obtained the chemical potential for bosons:

$$\beta\mu_0^i = \ln\big(1 - \exp[-n_i\lambda_i^2/\xi_i]\big). \tag{S73}$$

Exchange free energy

The first-order exchange contribution to the thermodynamic potential was given by[S8,S10]

$$\Omega_{\text{exch}}(\beta,\mu) = -\sum_{i=\text{e,h}} \frac{\xi_i}{2A} \sum_{\mathbf{k},\mathbf{p}} \frac{2\pi e^2}{4\pi\epsilon_0\epsilon|\mathbf{k}-\mathbf{p}|} f_i(k,\beta,\mu) f_i(p,\beta,\mu) \tag{S74}$$

where f_i is the Fermi-Dirac distribution for particle i and $\beta^{-1} = k_\mathrm{B}T$. This equation is exact when we use the chemical potential for the interacting system, μ. Following previous work,[S10,S11] we replaced this exact chemical potential by the one corresponding to the non-interacting system, Eq. (S68). Thus, our Helmholtz exchange free energy was

$$\mathcal{F}_\mathrm{exch}(\beta, n) = -\sum_{i=e,h} \frac{\xi_i}{2A} \sum_{\mathbf{k},\mathbf{p}} \frac{2\pi e^2}{4\pi\epsilon_0\epsilon|\mathbf{k}-\mathbf{p}|} f_i\big(k, \beta, \mu_0(n)\big) f_i\big(p, \beta, \mu_0(n)\big) \tag{S75}$$

Moving to dimensionless quantities, we let $k' = k/k_\mathrm{F}$ and $t = T/T_\mathrm{F}$, where the Fermi temperature is

$$T_\mathrm{F} = \frac{\hbar^2 k_\mathrm{F}^2}{2m_\mathrm{red}k_\mathrm{B}} \tag{S76}$$

In these quantities, the ideal chemical potentials became

$$\beta\mu_0^i = \begin{cases} \ln\left[\exp\left(\frac{1}{t(\sigma+1)}\right) - 1\right] & i = \mathrm{e} \\ \ln\left[\exp\left(\frac{\sigma}{t(\sigma+1)}\right) - 1\right] & i = \mathrm{h} \end{cases} \tag{S77}$$

and the Fermi-Dirac distributions become

$$f_i(k', t, n) = \begin{cases} \frac{\exp\left[1/\big(t(\sigma+1)\big)\right]-1}{\exp\left[k'^2/\big(t(\sigma+1)\big)\right]+\exp\left[1/\big(t(\sigma+1)\big)\right]-1} & i = e \\ \frac{\exp\left[\sigma/\big(t(\sigma+1)\big)\right]-1}{\exp\left[k'^2\sigma/\big(t(\sigma+1)\big)\right]+\exp\left[\sigma/\big(t(\sigma+1)\big)\right]-1} & i = h \end{cases} \tag{S78}$$

Note that when the electrons and holes have different masses, they experience two different effective temperatures:

$$t_i = \begin{cases} t(\sigma+1) & i = \mathrm{e} \\ t(\sigma+1)/\sigma & i = \mathrm{h} \end{cases} \tag{S79}$$

In these reduced units, the density dependence entered only through the reduced temperature.

Converting the sums to integrals,

$$\frac{\mathcal{F}_{\mathrm{exch}}(t)}{N} = -\sum_{i=\mathrm{e,h}} \frac{k_{\mathrm{F}} e^2}{(2\pi)^2} \int d^2k' \int d^2p' \frac{f_i(k',t) f_i(p',t)}{4\pi\epsilon_0\epsilon|\mathbf{k}' - \mathbf{p}'|} \tag{S80}$$

Phatisena and co-workers[S10] expressed the exchange free energy of a two-dimensional electron gas relative to its exchange energy at zero temperature, E_{exch}:

$$\frac{\mathcal{F}_{\mathrm{exch}}^{2DEG}(t)}{\mathcal{E}_{\mathrm{exch}}} = 3\int_0^\infty dx x^2 f(x,t) \int_0^1 dz f(xz,t) K(z) \tag{S81}$$

$$= C(t) \tag{S82}$$

where $K(z)$ is the complete elliptic integral of the first kind. To apply this result for our two-component system, we evaluated it at the previously defined effective temperatures:

$$F_{\mathrm{exch}} = -\sum_{i=\mathrm{e,h}} \frac{1.2004}{r_s} C(t_i) Ry_{\mathrm{ex}} \tag{S83}$$

Correlation free energy

Using the linked cluster theorem,[S12] the correlation thermodynamic potential for a bilayer electron-hole liquid obtained by summing ring diagrams[S8] was expressed as

$$\Omega_{\mathrm{corr}}(\beta,\mu) = -\frac{1}{2\beta}\sum_{\omega_l,k}\int_0^1 \frac{d\lambda}{\lambda}\begin{bmatrix}\Pi_{\mathrm{e}} & \Pi_{\mathrm{h}}\end{bmatrix}\begin{bmatrix} U_{\mathrm{ee}}(\lambda,k)U^0_{\mathrm{ee}}(\lambda,k) & U_{\mathrm{eh}}(\lambda,k)U^0_{\mathrm{eh}}(\lambda,k) \\ U_{\mathrm{eh}}(\lambda,k)U^0_{\mathrm{eh}}(\lambda,k) & U_{\mathrm{hh}}(\lambda,k)U^0_{\mathrm{hh}}(\lambda,k)\end{bmatrix}$$
$$\times\begin{bmatrix}\Pi_{\mathrm{e}}(k,\omega_l,\beta) \\ \Pi_{\mathrm{h}}(k,\omega_l,\beta)\end{bmatrix} \tag{S84}$$
$$= -\frac{A}{2\beta(2\pi)^2}\sum_{\omega_l}\int d^2k\int_0^1 d\lambda U^0_{\mathrm{ee}}\frac{\lambda U^0_{\mathrm{ee}}}{1-\lambda\big(\Pi_{\mathrm{e}}+\Pi_{\mathrm{h}}\big)U^0_{\mathrm{ee}}+\lambda^2\big(1-\exp[-4kd]\big)\Pi_{\mathrm{e}}\Pi_{\mathrm{h}}\big(U^0_{\mathrm{ee}}\big)^2}$$
$$\times\begin{bmatrix}\Pi_{\mathrm{e}} & \Pi_{\mathrm{h}}\end{bmatrix}\begin{bmatrix}1+\lambda\big(\exp[-4kd]-1\big)\Pi_{\mathrm{h}}U^0_{\mathrm{ee}} & \exp[-2kd] \\ \exp[-2kd] & 1+\lambda\big(\exp[-4kd]-1\big)\Pi_{\mathrm{e}}U^0_{\mathrm{ee}}\end{bmatrix}\begin{bmatrix}\Pi_{\mathrm{e}} \\ \Pi_{\mathrm{h}}\end{bmatrix} \tag{S85}$$

In lieu of integrating over a continuous frequency, we now summed over discrete bosonic Matsubara frequencies

$$\omega_l = \frac{2\pi l}{\beta} \quad l = 0, \pm 1, \pm 2, ... \tag{S86}$$

Additionally, the static ($l = 0$) and dynamic ($l \neq 0$) finite-temperature polarizabilities were[S10]

$$\Pi_j(k, l=0, t) = -\frac{4m_j}{\pi\hbar^2 k}\int_0^{k/2} dx \frac{x f(x,t_j)}{\sqrt{k^2-4x^2}} \tag{S87}$$

and

$$\Pi_j(k, l\neq 0, t) = -\frac{4m_j}{\pi\hbar^2}\int_0^{\infty} dx \frac{x f_j(x,t)\cos\phi}{\Big[\big(k^4-4x^2k^2-4l^2\pi^2t_j^2\big)^2+16l^2\pi^2t_j^2k^4\Big]^{1/4}} \tag{S88}$$

where

$$\tan(2\phi) = \frac{4k^2 l\pi t_j}{k^4-4x^2k^2-4l^2\pi^2t_j^2} \tag{S89}$$

When computing ϕ, we used "atan2(y,x)," the two-argument arctangent function which yields the angle between the positive x-axis and the point (x,y). Using the same dimensionless quantities as before,

$$\begin{aligned}
&F_{\text{corr}}(t,r_s) = -\frac{t}{r_s^2}\sum_{\omega_l}\int_0^\infty dk\int_0^1 d\lambda \\
&\times\Bigg\{\frac{k\lambda}{1-\lambda\big(\Pi_{\text{e}}+\Pi_{\text{h}}\big)U_{\text{ee}}^0+\lambda^2\big(1-\exp[-4\sqrt{2}kd'/r_s]\big)\Pi_{\text{e}}\Pi_{\text{h}}\big(U_{\text{ee}}^0\big)^2} \\
&\times\Big(\big(U_{\text{ee}}^0\big)^2\big(\Pi_{\text{e}}^2+\Pi_{\text{h}}^2\big)+2\big(U_{\text{ee}}^0\big)^2\Pi_{\text{e}}\Pi_{\text{h}}\exp[-2\sqrt{2}kd'/r_s] \\
&+\lambda\big(\exp[-4\sqrt{2}kd'/r_s]-1\big)\big(U_{\text{ee}}^0\big)^3\big(\Pi_{\text{e}}+\Pi_{\text{h}}\big)\Pi_{\text{e}}\Pi_{\text{h}}\Big)\Big)\Bigg\}Ry_{\text{ex}}
\end{aligned} \tag{S90}$$

Fitting exchange-correlation free energies

To obtain smooth exchange-correlation chemical potentials, we fit the exchange-correlation free energies $F_{\text{XC}} = F_{\text{exch}} + F_{\text{corr}}$ to a function which has a smooth and simple derivative. Our target function was

$$F_{\text{fit}}(n;n_{\text{C}}) = \begin{cases} \sum_{j=0}^{N} a_j n^{j/2} & n < n_{\text{C}} \\ \sum_{j=N+1}^{2N+2} a_j (\ln n)^{j-N-1} & n \geq n_{\text{C}} \end{cases} \tag{S91}$$

N is the order of both polynomials and n_{C} is the density cutoff (typically 10^9 cm^{-2}) above which we fit the data using a polynomial of $\ln n$. By defining

$$c_j(n;n_C) = \begin{cases} n^{j/2} & j \leq N \text{ and } n < n_C \\ 0 & j \leq N \text{ and } n \geq n_C \\ 0 & j > N \text{ and } n < n_C \\ (\ln n)^{j-N-1} & j > N \text{ and } n \geq n_C \end{cases} \tag{S92}$$

and $\vec{a}$ as our vector of desired fitting coefficients, Eq. S91 was written as

$$F_{\text{fit}}(n; n_{\text{C}}) = \vec{c}(n; n_{\text{C}}) \cdot \vec{a} \tag{S93}$$

At n_{C}, we required the values and first derivatives of the polynomials to the left and right to be equal:

$$\sum_{j=0}^{N} a_j (n_C)^{j/2} = \sum_{j=N+1}^{2N+2} a_j (\ln n_C)^{j-N-1} \tag{S94}$$

$$\frac{d}{dn}\left(\sum_{j=0}^{N} a_j (n)^{j/2}\right)_{n=n_C} = \frac{d}{dn}\left(\sum_{j=N+1}^{2N+2} a_j (\ln n)^{j-N-1}\right)_{n=n_C} \tag{S95}$$

By defining

$$d_{1j} = \begin{cases} n_C^{j/2} & j \leq N \\ -(\ln n_C)^{j-N-1} & j > N \end{cases} \tag{S96}$$

$$d_{2j} = \begin{cases} \frac{j}{2} n_{\text{C}}^{j/2-1} & j \leq N \\ -\frac{(j-N-1)}{n_{\text{C}}}(\ln n_{\text{C}})^{j-N-2} & j > N \end{cases} \tag{S97}$$

our constraints became

$$\vec{d}_1 \cdot \vec{a} = 0 \tag{S98}$$

$$\vec{d}_2 \cdot \vec{a} = 0 \tag{S99}$$

To minimize the squared residual error

$$\mathcal{E} = \sum_{\alpha=1}^{N_{\text{density}}} \left(F_{\text{fit}}(n_\alpha; n_C) - F_{\text{XC}}(n_\alpha)\right)^2 \tag{S100}$$

subject to our constraints, we introduced two Lagrange multipliers, λ_1 and λ_2. Our Lagrangian was

$$\mathcal{L}(\{n_\alpha\}, n_{\text{C}}, \lambda_1, \lambda_2) = \sum_{\alpha=1}^{N_{\text{density}}} \left(F_{\text{fit}}(n_\alpha; n_{\text{C}}) - F_{\text{XC}}(n_\alpha)\right)^2 - \lambda_1 \vec{d}_1 \cdot \vec{a} - \lambda_2 \vec{d}_2 \cdot \vec{a} \tag{S101}$$

To find $\vec{a}$ which minimizes $\mathcal{L}$, we set its derivative equal to 0:

$$\frac{\partial \mathcal{L}}{\partial a_i} = 0 \tag{S102}$$

$$= 2\sum_{\alpha=1}^{N_{\text{density}}} \left(F_{fit}(n_\alpha; n_C) - F_{XC}(n_\alpha)\right)^2\Big) \left.\frac{\partial F_{fit}}{\partial a_i}\right|_{n=n_\alpha} - \lambda_1 d_{1i} - \lambda_2 d_{2i} \tag{S103}$$

Note that

$$\left.\frac{\partial F_{\text{fit}}}{\partial a_i}\right|_{n=n_\alpha} = c_i(n_\alpha; n_{\text{C}}) \tag{S104}$$

Defining

$$\chi_{ij}^{-1} = 2\sum_{\alpha=1}^{N_{\text{density}}} c_i(n_\alpha; n_{\text{C}}) c_j(n_\alpha; n_{\text{C}}) \tag{S105}$$

and

$$b_i = 2\sum_{\alpha=1}^{N_{\text{density}}} F_{\text{XC}}(n_\alpha) c_i(n_\alpha) \tag{S106}$$

(S103) became

$$\chi^{-1} \cdot \vec{a} = \vec{b} + \lambda_1 \vec{d_1} + \lambda_2 \vec{d_2} \tag{S107}$$

Our desired coefficients were given by

$$\vec{a} = \chi \cdot \vec{b} + \lambda_1 \chi \cdot \vec{d_1} + \lambda_2 \chi \cdot \vec{d_2} \tag{S108}$$

The Lagrange multipliers were determined by

$$0 = \vec{d_1} \cdot \vec{a} \tag{S109}$$

$$= \vec{d_1} \cdot \chi \cdot \vec{b} + \lambda_1 \vec{d_1} \cdot \chi \cdot \vec{d_1} + \lambda_2 \vec{d_1} \cdot \chi \cdot \vec{d_2} \tag{S110}$$

$$0 = \vec{d_2} \cdot \vec{a} \tag{S111}$$

$$= \vec{d_2} \cdot \chi \cdot \vec{b} + \lambda_1 \vec{d_2} \cdot \chi \cdot \vec{d_1} + \lambda_2 \vec{d_2} \cdot \chi \cdot \vec{d_2} \tag{S112}$$

By defining

$$g_{ij} = \vec{d}_i \cdot \chi \cdot \vec{d}_j \tag{S113}$$

and

$$h_i = -\vec{d}_i \cdot \chi \cdot \vec{b} \tag{S114}$$

we have

$$G \cdot \vec{\lambda} = \vec{h} \tag{S115}$$

so

$$\vec{\lambda} = G^{-1} \cdot \vec{h} \tag{S116}$$

Figure S3 shows the results of the fit compared to the raw data for a bilayer EHL with $\sigma = 0.1$, $d = 1.5a_{\mathrm{ex}}$, and $T = 8\mathrm{K}$. The deviation is at most 0.02 Ry_{ex}.

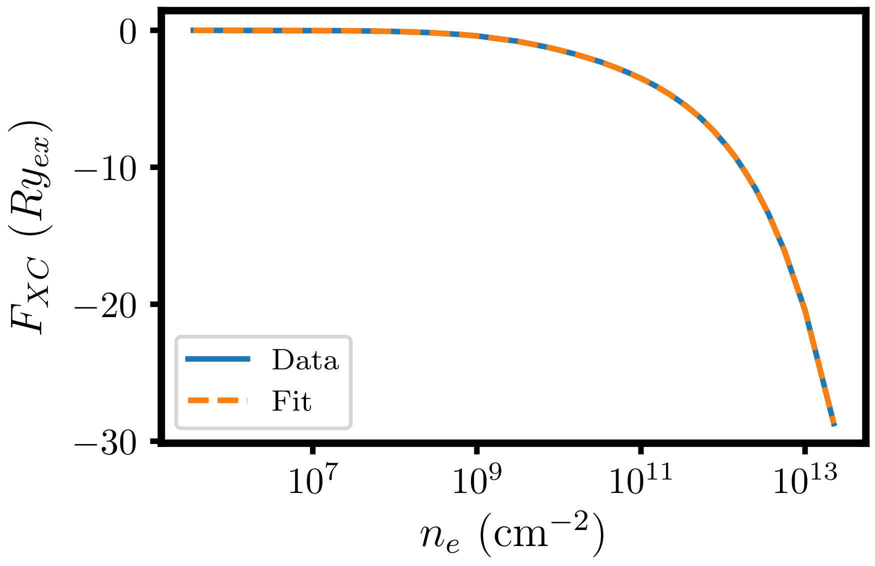

Figure S3: Exchange and correlation contributions to the Helmholtz free energy of a bilayer EHL with $\sigma = 0.1$, $d = 1.5a_{\mathrm{ex}}$, and $T = 8\mathrm{K}$. The x-axis is the density of free carriers in units of number of electrons per cm^2.

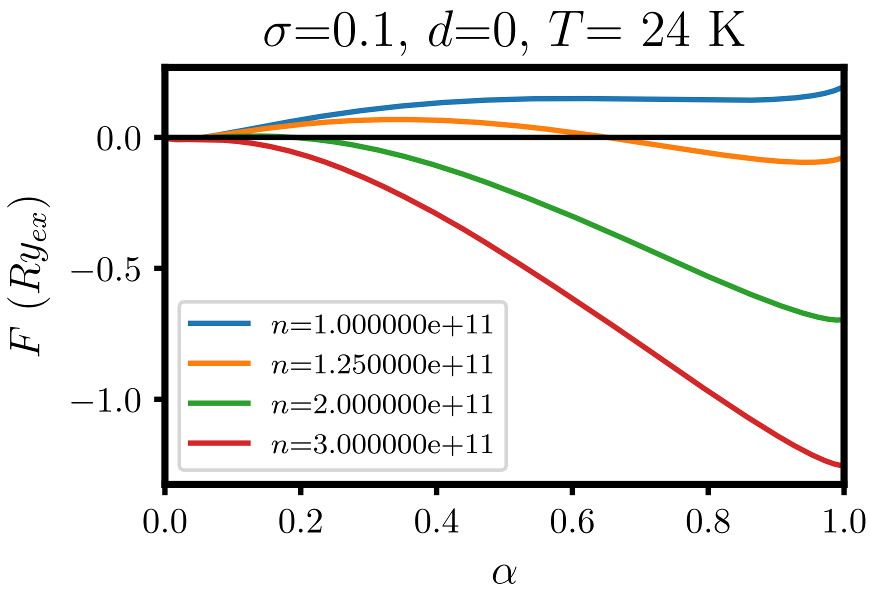

Figure S4: Helmholtz free energy as a function of ionization ratio. The total excitation densities in the legend are in units of number of electrons per cm^2. The Mott density is $n_{\mathrm{Mott}} \lesssim 1.25 \times 10^{11}$ cm^{-2}.

Solving the law of mass action

To evaluate the ionization ratio α at low densities, we solved the law of mass action given by Eqs. 11 and 12 in the main text using a root-finding algorithm: the bisection method. However, multiple roots appeared at higher densities (shown below). The correct choice was the one that minimizes the system's free energy, so we first computed the change in Helmholtz free energy due to carriers partitioning into free and bound states:

$$dF = \mu_{\mathrm{eh}} dn_{\mathrm{eh}} + \mu_{\mathrm{X}} dn_{\mathrm{X}} \tag{S117}$$

$$= (\mu_{\mathrm{eh}} - \mu_{\mathrm{X}}) n_{\mathrm{tot}} d\alpha. \tag{S118}$$

Thus,

$$\frac{dF}{d\alpha} = (\mu_{\mathrm{eh}} - \mu_{\mathrm{X}}) n_{\mathrm{tot}} \tag{S119}$$

Integrating from some arbitrary reference value α_0 leads to

$$F(\alpha) = F(\alpha_0) + n_{tot} \int_{\alpha_0}^{\alpha} \left(\mu_{tot}^{free}(\tilde{\alpha}) - \mu_{tot}^{X}(\tilde{\alpha})\right) d\tilde{\alpha} \tag{S120}$$

Because the location of the minimum doesn't depend on $F(\alpha_0)$ or the factor of n_{tot}, these terms were neglected.

Fig. S5 shows the Helmholtz free energy as a function of the ionization ratio α. When the total excitation density is 1.25 $\times 10^{11}$ cm^{-2}, there are two extrema located at $\alpha \approx$0.35 and 0.95. While these are both solutions to the law of mass action, $\alpha \approx 0.95$ is the correct choice as it minimizes the free energy.

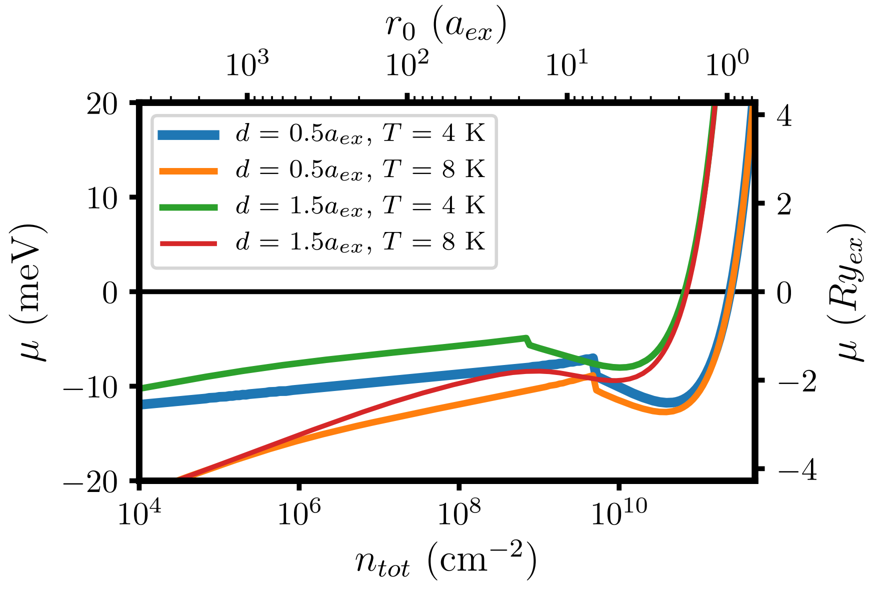

Figure S5: Chemical potentials for the electron-hole-exciton system with $\sigma = 0.1$.

Maxwell equal-area constructions

After calculating the ionization ratio α as a function of $n_{\rm tot}$, we obtained chemical potentials for the electron-hole-exciton system shown in Fig. S5. These potentials violate thermodynamic stability for densities near 10^{10} cm^{-2} since their first derivatives with respect to density are negative. We determined the liquid-gas coexistence densities using a standard Maxwell

equal-area construction;[S13] we identified the value of the chemical potential μ_{constant} that encloses two regions of equal area.

References

S1. Szabo, A.; Ostlund, N. S. *Modern Quantum Chemistry*; Dover Publications, Inc.: New York, 1996; pp 153-159, 231-241.

S2. Dennis, J. E.; Schnabel, R. B. *Numerical Methods for Unconstrained Optimization*; SIAM: Philadelphia, 1996; pp 198-203.

S3. Dunning, T. H. Gaussian Basis Sets for Use in Correlated Molecular Calculations. I. The Atoms Boron through Neon and Hydrogen. *J. Chem. Phys.* **1989**, *90* (2), 1007-1023.

S4. Bernard, E. P.; Krauth, W. Two-Step Melting in Two Dimensions: First-Order Liquid-Hexatic Transition. *Phys. Rev. Lett.* **2011**, *107* (15), 155704.

S5. Frenkel, D.; Smit, B. *Understanding Molecular Simulation*; Academic Press: San Diego, 2002; pp 37-39.

S6. Brinkman, W. F.; Rice, T. M. Electron-Hole Liquids in Semiconductors. *Phys. Rev. B* **1972**, *7* (4), 1508-1523.

S7. Kuramoto, Y.; Kamimura, H. Theory of Two-Dimensional Electron-Hole Liquids. *J. Phys. Soc. Jpn.* **1974**, *37* (3), 716-723.

S8. Fetter, A. L.; Walecka, J. D. *Quantum Theory of Many-Particle Systems*; Dover Publications, Inc.: New York, 2003; pp 21-30, 151-167, 267-289.

S9. Inagaki, A.; Katayama, S. Binding Energy of the Electron-Hole Liquid in Type-II $(GaAs)_m/(AlAs)_m$ Quantum Wells. *J. Phys. Soc. Jpn.* **2003**, *72* (6), 1452-1457.

S10. Phatisena, S.; Amritkar, R. E.; Panat, P. V. Exchange and Correlation Potential for a Two-Dimensional Electron Gas at Finite Temperatures. *Phys. Rev. A* **1986**, *34* (6), 5070-5079.

S11. Rustagi, A.; Kemper, A. F. Theoretical Phase Diagram for the Room-Temperature Electron-Hole Liquid in Photoexcited Quasi-Two-Dimensional Monolayer MoS_2. *Nano Lett.* **2018**, *18* (1), 455-459.

S12. Mahan, G. D. *Many-Particle Physics*; Springer Science+Business Media: New York, 2000; pp 142-154.

S13. Chandler, D. *Introduction to Modern Statistical Mechanics*; Oxford University Press: New York, 1987; pp 42-44.
